# Building better enzymes: Molecular basis of improved non‐natural nucleobase incorporation by an evolved DNA polymerase

**DOI:** 10.1002/pro.3762

**Published:** 2019-11-14

**Authors:** Zahra Ouaray, Isha Singh, Millie M. Georgiadis, Nigel G. J. Richards

**Affiliations:** ^1^ School of Chemistry Cardiff University Cardiff UK; ^2^ Department of Biochemistry & Molecular Biology Indiana University School of Medicine Indianapolis Indiana

**Keywords:** DNA replication, enzyme engineering, expanded genetic alphabets, molecular dynamics, polymerase

## Abstract

Obtaining semisynthetic microorganisms that exploit the information density of “hachimoji” DNA requires access to engineered DNA polymerases. A KlenTaq variant has been reported that incorporates the “hachimoji” **P**:**Z** nucleobase pair with a similar efficiency to that seen for Watson–Crick nucleobase incorporation by the wild type (WT) KlenTaq DNA polymerase. The variant polymerase differs from WT KlenTaq by only four amino acid substitutions, none of which are located within the active site. We now report molecular dynamics (MD) simulations on a series of binary complexes aimed at elucidating the contributions of the four amino acid substitutions to altered catalytic activity. These simulations suggest that WT KlenTaq is insufficiently flexible to be able to bind AEGIS DNA correctly, leading to the loss of key protein/DNA interactions needed to position the binary complex for efficient incorporation of the “hachimoji” **Z** nucleobase. In addition, we test literature hypotheses about the functional roles of each amino acid substitution and provide a molecular description of how individual residue changes contribute to the improved activity of the KlenTaq variant. We demonstrate that MD simulations have a clear role to play in systematically screening DNA polymerase variants capable of incorporating different types of nonnatural nucleobases thereby limiting the number that need to be characterized by experiment. It is now possible to build DNA molecules containing nonnatural nucleobase pairs in addition to A:T and G:C. Exploiting this development in synthetic biology requires engineered DNA polymerases that can replicate nonnatural nucleobase pairs. Computational studies on a DNA polymerase variant reveal how amino acid substitutions outside of the active site yield an enzyme that replicates nonnatural nucleobase pairs with high efficiency. This work will facilitate efforts to obtain bacteria possessing an expanded genetic alphabet.

**Abbreviations**
AEGIS: artificially expanded genetic information systems
DCCM: dynamic cross‐correlation map
MD: molecular dynamics
**P**: 2‐amino‐8‐(1‐beta‐D‐2′‐deoxyribofuranosyl)imidazo [1,2‐a]‐1,3,5‐triazin‐[8H]‐4‐one
PCA: principal component analysis
PDB: Protein Data Bank
RMSD: root mean square deviation
WT: wild type
**Z**: 6‐amino‐3‐(2′‐deoxyribofuranosyl)‐5‐nitro‐1H‐pyridin‐2‐one

## 1 INTRODUCTION

**Figure 1:**
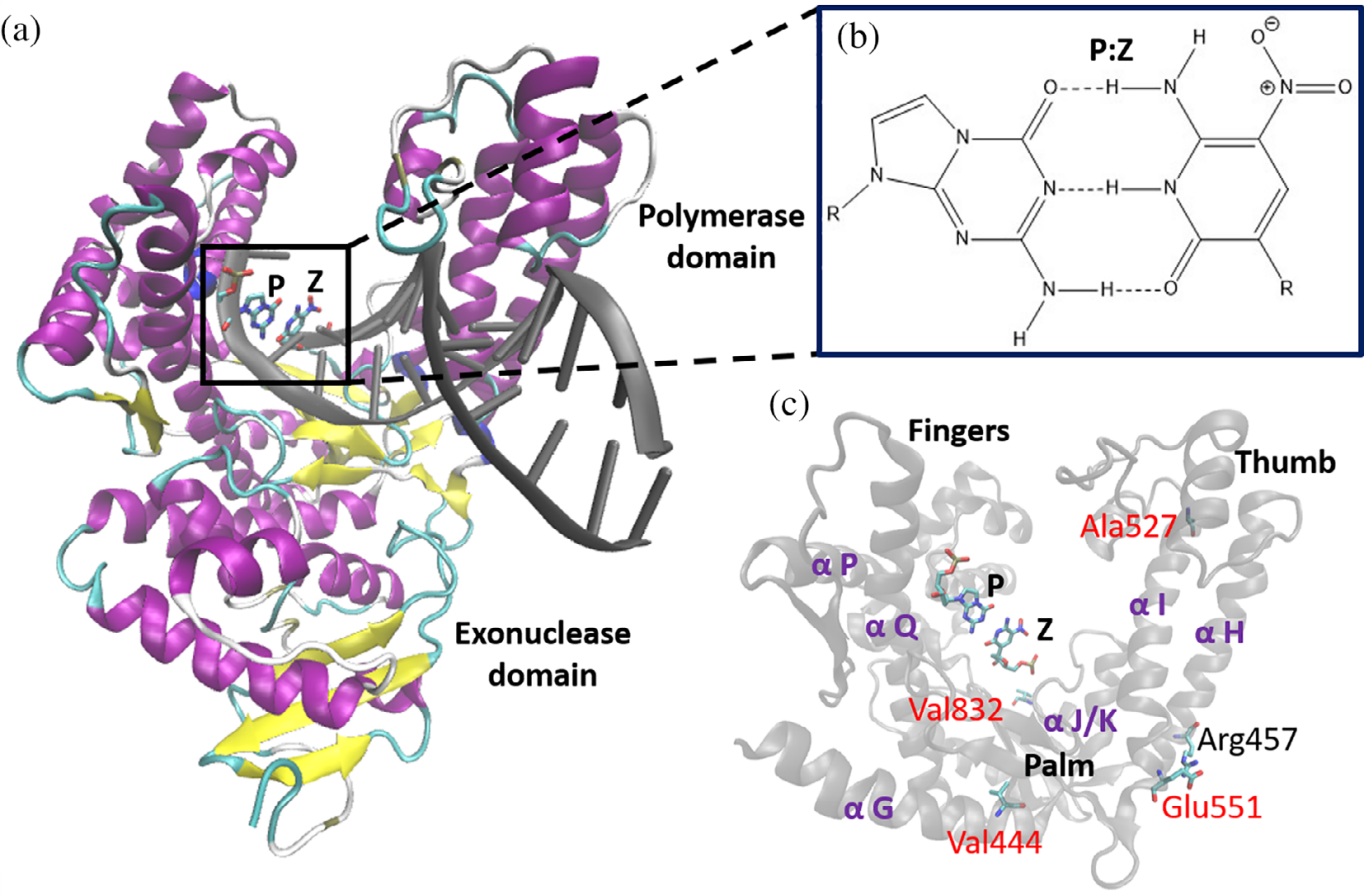
(a) Illustration of the X‐ray crystal structure of the “evolved” variant KlenTaq polymerase in its binary complex (PDB: http://firstglance.jmol.org/fg.htm?mol=5W6Q),^24^ showing the location of the **P**:**Z** nucleobase pair in the polymerase domain. Helices and strands are colored purple and yellow, respectively. (b) Chemical structure of the **P**:**Z** nucleobase pair. R indicates the location of the 2'‐deoxyribose substituent and hydrogen bonds are indicated by dashed lines. (c) Close‐up of the polymerase domain showing the palm, fingers and thumb domains, the helical regions ***αG*‐*αK***, ***αP***, and ***αQ***, and the side chains of the mutated residues, Val444, Ala527, Glu551, and Val832, in the variant KlenTaq polymerase

The development of “semisynthetic” microorganisms possessing artificially expanded genetic information systems (AEGIS) will permit access to cells with novel phenotypes and biotechnological applications.^1^, ^2^, ^3^, ^4^ Nonnatural nucleobase pairs that meet the size and/or hydrogen bonding complementarity rules of Watson–Crick base pairing have been described over the past two decades (Figure S1A),^5^, ^6^, ^7^, ^8^ including the complementary 2‐amino‐8‐(1‐beta‐D‐2′‐deoxyribofuranosyl)imidazo [1,2‐a]‐1,3,5‐triazin‐[8H]‐4‐one (trivially known as **P**) and 6‐amino‐3‐(2′‐deoxyribofuranosyl)‐5‐nitro‐1H‐pyridin‐2‐one (trivially known as **Z**) nucleobase pair that is present in “hachimoji” DNA (Figure 1).^1^, ^5^, ^9^, ^10^, ^11^, ^12^ As is true of naturally occurring (Watson–Crick) DNA, AEGIS DNA duplexes containing **P**:**Z** pairs interconvert easily between A‐ and B‐helical forms.^13^, ^14^ In addition, B‐form DNA tolerates the inclusion of multiple consecutive **P**:**Z** nucleobase pairs with minimal structural impact on the double helix when compared to duplexes containing only A:T or G:C base pairs.^9^

Identifying DNA polymerases capable of catalyzing the incorporation of **P**:**Z** nucleobase pairs with efficiencies comparable to those that replicate Watson–Crick DNA is a necessary prerequisite to realizing the promise of expanded genetic alphabets.^15^, ^16^ A variety of library generation and selection strategies have been developed to re‐engineer the fidelity of DNA polymerases,^17^, ^18^, ^19^ including the large (Klenow) fragment of *Thermus aquaticus* DNA polymerase I, which lacks the N‐terminal 5′‐3′ exonuclease domain (KlenTaq).^20^ Thus, a compartmentalized self‐replication strategy^21^, ^22^ was used to obtain a KlenTaq variant capable of incorporating d**Z**TP opposite a **P** nucleobase in the template strand with a greatly improved efficiency relative to the wild type (WT) precursor (Figure 1).^23^ The evolved KlenTaq variant contains four amino acid replacements (M444 V, P527A, D551E, and E832V; Figure 1), all of which are distal to the active site. As a result, none of these residues interact directly with either the primer/template **P:Z** in the active site or with incoming nucleotide triphosphate (**dZTP**) in high resolution crystal structures of the pre‐ and post‐incorporation complexes for this variant polymerase (http://firstglance.jmol.org/fg.htm?mol=PDB:5W6K and http://firstglance.jmol.org/fg.htm?mol=PDB:5W6Q, respectively).^24^

In considering, how this evolved KlenTaq variant might differ from WT KlenTaq, Singh et al. analyzed differences in relative domain motions in the unnatural complexes as compared to the natural complexes.^24^ There were no significant differences between the relative domain positions in the crystal structures of pre‐incorporation complexes for the Watson–Crick (WT KlenTaq within incoming dNTP) and AEGIS (KlenTaq variant with incoming d**Z**TP paired to template **P**) systems. In contrast, there were significant differences in the post‐incorporation Watson–Crick (WT KlenTaq with G:C bound in the active site) versus AEGIS (evolved KlenTaq with **P:Z** bound in the active site) complexes. In forming the ternary pre‐incorporation complex, there is a large motion of the fingers domain as it closes down on the incoming dNTP when compared to the binary post‐incorporation complex.^24^ Thus, the average rotation angle calculated from a comparison of the pre‐ and post‐incorporation AEGIS complexes was 63.6° while that for the Watson–Crick complexes was decreased by 4–5° despite the fact that the Watson–Crick and AEGIS pre‐incorporation complexes ultimately reached a similar structural state. Modeling the AEGIS template/primer bound to WT KlenTaq in a post‐incorporation complex resulted in two significant “clashes,” suggesting that the structure of the AEGIS template‐primer differed significantly from that of its Watson–Crick counterpart. Collectively, the crystallographic evidence supported the hypothesis that the four amino acid substitutions in the KlenTaq variant led to increased flexibility in the enzyme and that this property was required for efficient incorporation of d**Z**TP opposite template **P**.^24^ In addition, this prior study showed that the post‐incorporation complex was the most affected by the residue substitutions. We now report a series of molecular dynamics (MD) simulations on eight binary complexes that not only test the idea that amino acid substitutions in the KlenTaq variant give rise to increased flexibility but also reveal how altered dynamical motions might contribute to the increased efficiency of **P**:**Z** nucleobase incorporation by the KlenTaq variant. These calculations set the scene for obtaining novel KlenTaq polymerase variants that can incorporate multiple types of nonnatural nucleobase pairs using MD simulations as part of rational, structure‐based strategies.

## 2 RESULTS AND DISCUSSION

### 2.1 WT KlenTaq and the evolved KlenTaq polymerase have similar dynamical properties in the absence of DNA

MD simulations of WT KlenTaq and the KlenTaq variant in water show that there is little difference in the dynamics of the two enzymes in the absence of bound DNA (Figures S2–S4). The similarity of motions in the “thumb,” “palm,” and “fingers” domains for both polymerases is evident from principal component analysis (PCA).^25^ Thus, projecting the simulation snapshots along the largest components (PC1 and PC2) shows that WT KlenTaq and the KlenTaq variant explore the same phase space (Figure S4).

### 2.2 WT KlenTaq polymerase binds differently to Watson–Crick and AEGIS DNA template/primer duplexes

We tested the hypothesis that the four amino acid substitutions in the KlenTaq variant exert their effects by modifying dynamical motions in the binary complex^21^ using four MD simulations: WT KlenTaq bound to Watson–Crick DNA (template: 5'‐AAAGGGCGCCGTGGTC‐3′/primer: 5'‐GACCACGGCGCC‐3′) and **P**:**Z**‐containing DNA (template: 5'‐AAAG**P**GCGCCGTGGTC‐3′/primer: 5'‐GACCACGGCGC**Z**‐3′) duplexes, and the KlenTaq variant bound to the same Watson–Crick and AEGIS DNA duplexes. Template and primer strands were positioned in these models based on the X‐ray crystal structure of the variant KlenTaq/AEGIS DNA binary complex.^24^ All four models were stable throughout the MD simulations based on root mean square deviation (RMSD) values (Figure S2). Most of the mobile residues were located in the fingers and thumb domains. The thumb domains in both polymerases when in the binary complexes, however, were less flexible when compared the cognate, uncomplexed enzymes (Figure S3).

**Figure 2:**
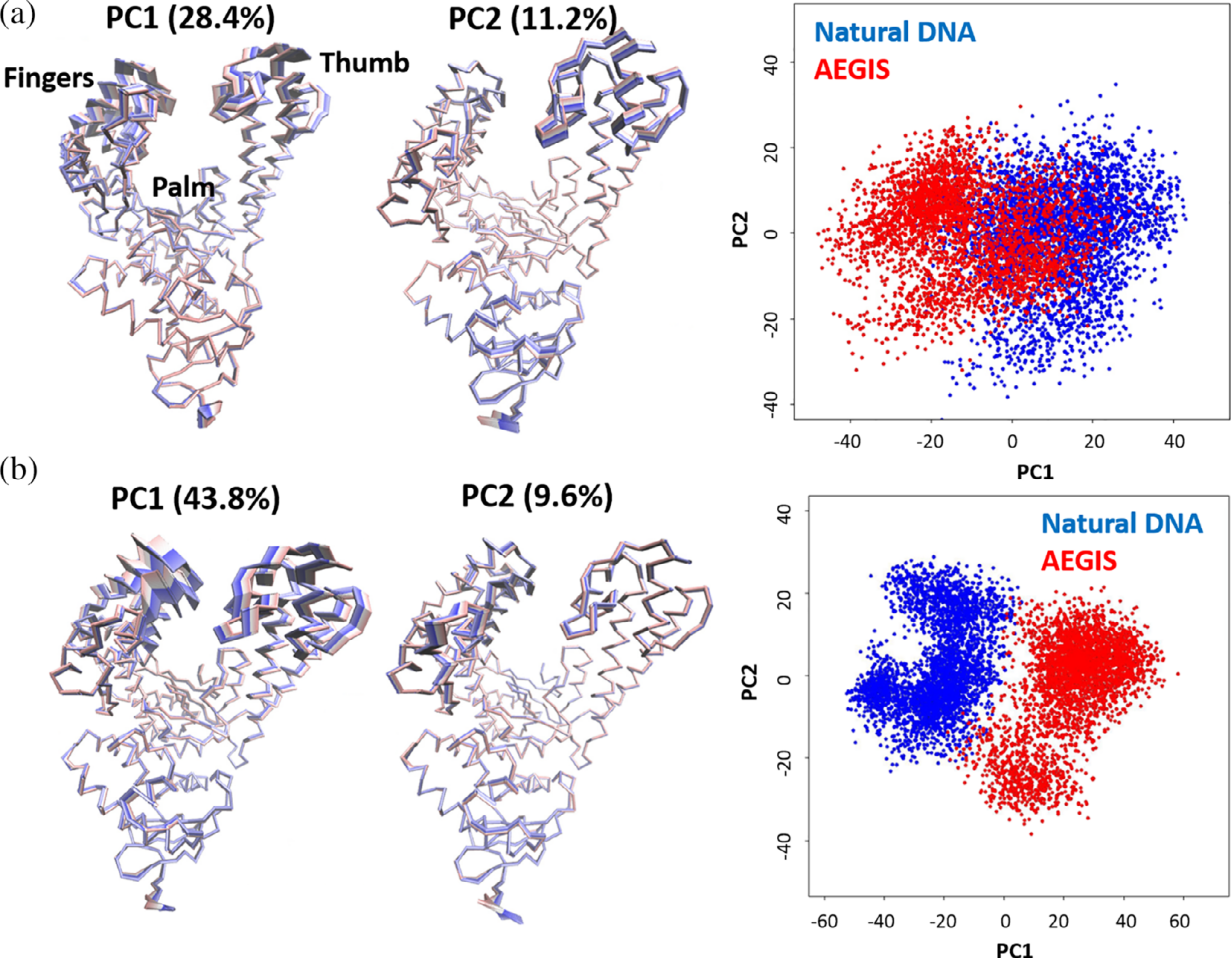
(a) Illustration of the motions associated with the two principal components PC1 (left) and PC2 (center) describing the variance of the WT polymerase/Watson–Crick DNA and WT polymerase/AEGIS DNA binary complexes at early (red), intermediate (white), and late (blue) stages of the MD simulations; (right) projection of trajectory snapshots along PC1 and PC2 for the WT polymerase/Watson–Crick DNA (blue) and WT polymerase/AEGIS DNA (red) binary complexes. (b) Illustration of the motions associated with the two principal components PC1 (left) and PC2 (center) describing the variance of the variant polymerase/Watson–Crick DNA and variant polymerase/AEGIS DNA binary complexes at early (red), intermediate (white), and late (blue) stages of the MD simulations; (right) Projection of trajectory snapshots along PC1 and PC2 for the variant polymerase/Watson–Crick DNA (blue) and variant polymerase/AEGIS DNA (red) binary complexes

The dynamical motions of both the protein and the DNA in the four trajectories were analyzed to understand how the altered residues (M444 V, P527A, D551E, and E832V) in the KlenTaq variant might impact incorporation of both Watson–Crick and AEGIS nucleobases. The trajectories of the binary complexes in which WT KlenTaq was bound to either Watson–Crick or AEGIS DNA gave two main principal components, which captured 39.6% of the coordinate variance (Figure 2a). The largest of these, PC1, is associated with a motion in which the thumb and fingers domains move away from each other in the binary complex. The second major component, PC2, describes local motions in the thumb domain. Projecting structures sampled in the two MD simulations along PC1 and PC2 (Figure 2a) shows that the two binary complexes separate along PC1, although they can sample common regions of phase space.

**Figure 3:**
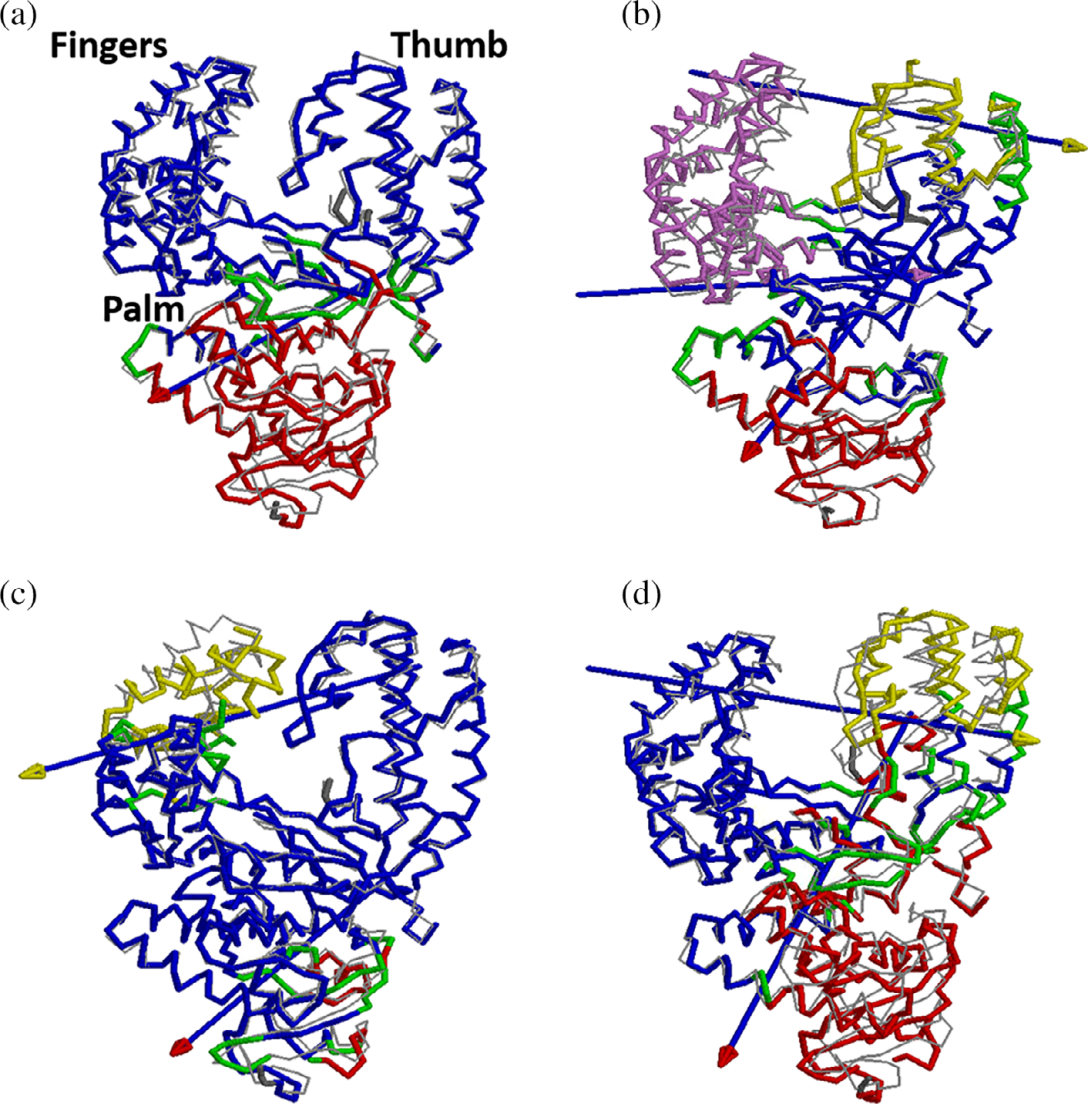
DynDom^26^ analysis of the four polymerase/DNA complexes. For reference, WT polymerase/Watson–Crick DNA binary complex (PDB: http://firstglance.jmol.org/fg.htm?mol=3SZ2)^30^ is shown in gray in all the panels. The axis of motion in each panel is indicated by an arrow, the head of which is colored to represent the moving domain (red, yellow, or purple). The fixed domain (blue) and regions that bend during the motion are shown in green. (a) C_α_ trace of the WT KlenTaq/Watson–Crick DNA binary complex. The exonuclease domain (red) moves away from the fingers, palm, and thumb domains. (b) C_α_ trace of the WT KlenTaq/AEGIS DNA binary complex. In this case, the fingers domain (purple) moves back into the plane of this image (purple headed arrow), the thumb domain (yellow) moves away from the fingers domain to the right, and the exonuclease domain (red) moves away from fingers, palm, and thumb domains as for the WT KlenTaq/Watson–Crick DNA binary complex. (c) C_α_ trace of the variant polymerase/Watson–Crick DNA binary complex. The tip of the fingers domain (yellow) moves to the left, away from the thumb domain. The exonuclease domain exhibits minimal movement in contrast to the other complexes. (d) C_α_ trace of the variant polymerase/AEGIS DNA binary complex. The thumb domain (yellow) moves away from the fingers domain; the exonuclease domain (red) moves away from the other domains

**Figure 4:**
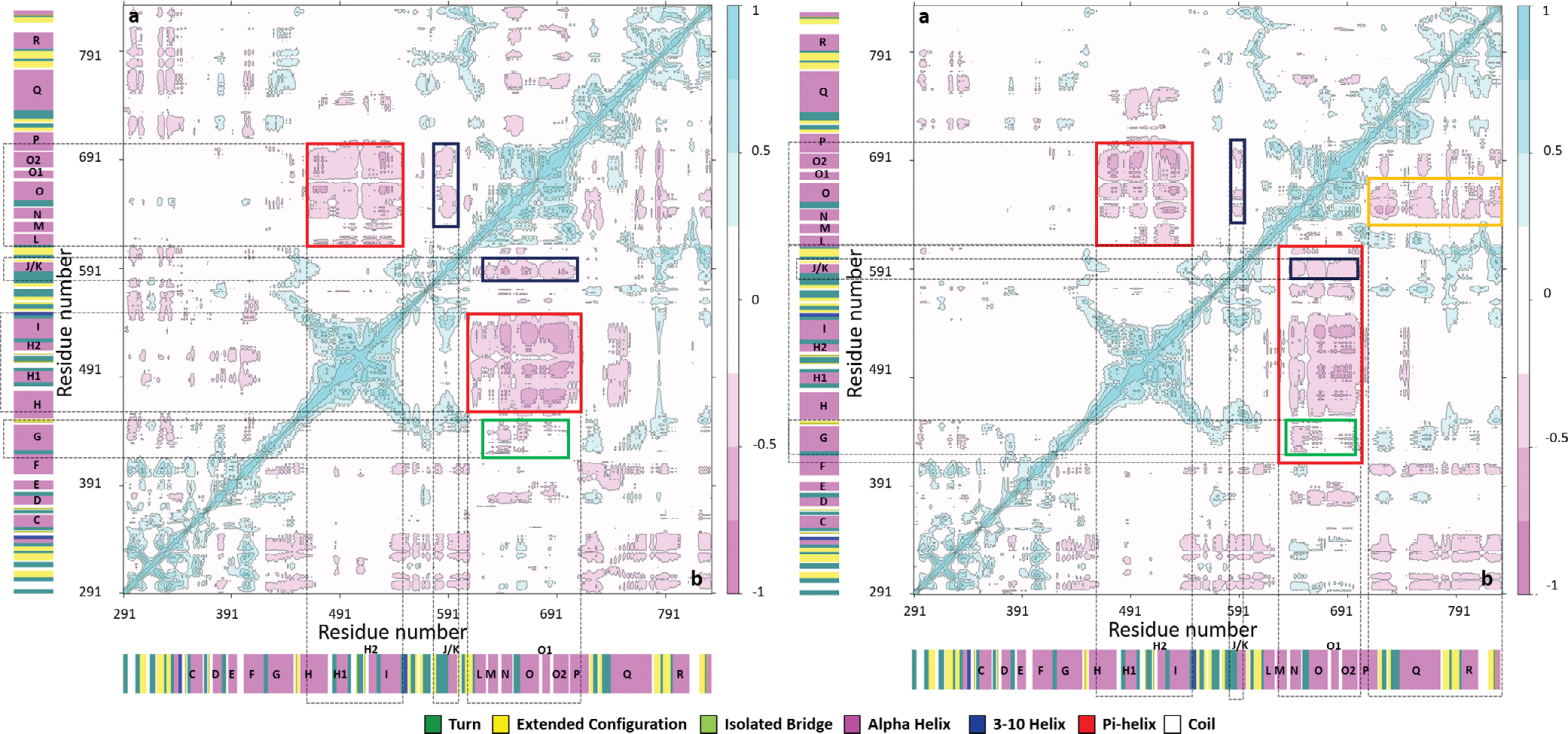
Dynamical cross correlation maps (DCCMs) computed for the four polymerase/DNA binary complexes; (left/top triangle) WT/Watson–Crick DNA binary complex, (left/bottom triangle) WT/AEGIS DNA binary complex, (right/top triangle) variant/Watson–Crick DNA binary complex, (right/bottom triangle) variant/AEGIS DNA binary complex. Correlated (range: 0.25 to 1) and anti‐correlated (range: −0.25 to −1) motions are colored from light to dark blue and pink, respectively. Areas rendered in white correspond to non‐correlated motions (range: −0.25 to 0.25). Secondary structural elements are also included on the map. See text for details

Domain motions (DynDom)^26^ computed from the MD trajectories highlight these differences in the dynamics of WT polymerase when complexed with either Watson–Crick or AEGIS DNA duplexes (Figure 3). In the WT KlenTaq/Watson–Crick DNA binary complex, the inactive exonuclease domain (residues 290–423) moves away from the polymerase domain (424–832) due to bending of residues in the palm (424–449, 553–614, and 774–832) (Figure 3a, Table S1). This motion is consistent with the need for the polymerase to change its conformation in order to bind the DNA duplex. Additional evidence is provided from the dynamic cross‐correlation map (DCCM)^27^ of this binary complex, which shows the motions of the fingers and thumb domains to be anti‐correlated (red box in Figure 4 [left/top]). An anti‐correlated motion of residues in the ***αJ/K*** helix of the palm domain (Figure 1) and fingers domain (blue box in Figure 4 [left/top]), which is not seen in the MD trajectory of the uncomplexed WT polymerase (Figure S4c), also supports the DynDom analysis.

The motional properties of the binary complex in which WT KlenTaq binds AEGIS DNA differ in a number of important aspects from that containing Watson–Crick DNA. For example, the domain motions are more complicated even though the exonuclease and polymerase domains still move apart (Figure 3b). Not only do the fingers and thumb move away from each other but the tip of the thumb about the AEGIS DNA becomes repositioned due to residues 477–543 moving away from the fingers (Figure 3b) with a rotation of 6.4° and a translation of 0.2 Å. In addition, the base of the fingers domain moves toward the thumb with a rotation of 7.5° and a translation of 0.2 Å (Table S1) due to bending of residues located in the palm, thereby also causing the tip of the fingers domain to move away from the thumb (Figure 3b). In agreement with these observations, the DCCM for the WT KlenTaq/AEGIS DNA binary complex shows anti‐correlated motions of (a) the fingers and thumb (red box in Figure 4 [left/bottom]) and (b) residues in the ***αJ/K*** helix of the palm domain with the fingers (blue box in Figure 4 [left/bottom]). Neither of these motions is seen for the uncomplexed WT polymerase (Figure S4c). The presence of the **Z**:**P** nucleobase pair also leads to anti‐correlated residue motions in the αG helix and the fingers domain (green box in Figure 4 [left/bottom]).

### 2.3 The evolved KlenTaq polymerase accommodates both Watson–Crick and AEGIS DNA template/primer duplexes albeit through distinct dynamical motions

While it is important for the evolved polymerase to recognize the unnatural AEGIS substrate, it must also recognize Watson–Crick nucleobases to be useful for the efficient replication of hachimoji DNA. Two major principal components capture 53.4% of the coordinate variance in the MD trajectories of the binary complexes of the KlenTaq variant bound to either Watson–Crick or AEGIS DNA. Projecting structures sampled in the two MD simulations along PC1 and PC2 shows a clear separation along both components, meaning that the two complexes sample different regions of phase space (Figure 2b). Comparing this projection for the KlenTaq variant (Figure 2b) with that for the binary complexes of WT enzyme (Figure 2a) shows an increased amplitude of the motions along PC1, supporting the idea that the residue substitutions confer increased flexibility onto the KlenTaq variant.

DynDom analysis suggests that the fingers domain of the variant polymerase exhibits different motions when binding Watson–Crick rather than AEGIS DNA. In addition, the tip of the fingers domain is tilted toward the base with a 14.6° rotation and a 0.5 Å translation in the KlenTaq variant/Watson–Crick DNA binary complex (Table S1), and the domain motions differ from those present in the KlenTaq variant/AEGIS DNA complex (Figure 3c,d). These findings are consistent with inferences based on comparing the X‐ray crystal structures of the KlenTaq variant/AEGIS DNA and the WT KlenTaq/Watson–Crick DNA binary complexes.^24^ The rotation of the thumb domain about residues 468–534 in the MD trajectory of the KlenTaq variant/AEGIS DNA binary complex is larger than that seen in the X‐ray structure (Table S1), perhaps as a consequence of replacing Pro‐527 by alanine. The exonuclease domain also moves away from the polymerase domain in the KlenTaq variant/AEGIS DNA binary complex, which is not seen when the variant polymerase binds to Watson–Crick DNA (Figure 3c,d). Finally, an anti‐correlated motion between the ***αJ/K*** helix in the palm and the fingers is present when the KlenTaq variant binds AEGIS DNA [blue box in Figure 4 [right/bottom]) that is absent in the free polymerase. In contrast, this anti‐correlated motion is decreased in the KlenTaq variant/Watson–Crick DNA binary complex. We conclude that the KlenTaq variant exhibits altered dynamical motions in binding the Watson–Crick DNA duplex, as compared to AEGIS DNA, even though it still readily accommodates this substrate.

### 2.4 Z:P‐containing DNA in the binary complex is sub‐optimally recognized by the WT KlenTaq polymerase

**Figure 5:**
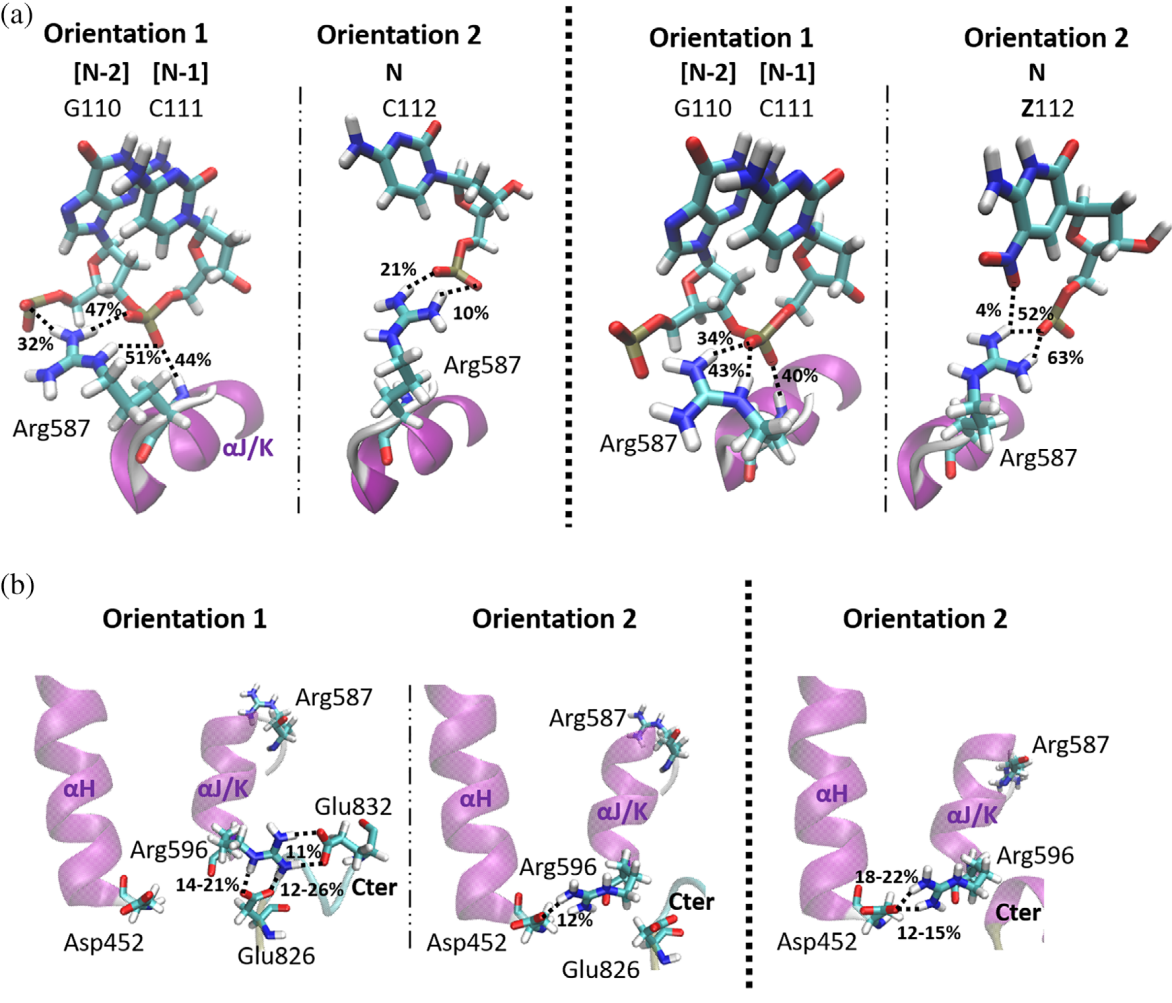
(a) Orientational preferences of Arg‐587 in the trajectories of the binary complexes between WT KlenTaq and Watson–Crick (left) and AEGIS (right) DNA. (b) Orientational preferences of the Arg‐596 side chain in trajectories of the WT KlenTaq/Watson–Crick (left/middle) and variant KlenTaq/AEGIS (right) binary complexes. The ***αH*** and ***αJ/K*** helices and the C‐terminal (Cter) region are shown in an illustration (purple). Atoms in named residues are colored according to the scheme: C, cyan; H, white; N, blue; O, red. Hydrogen bonds seen in the trajectories are represented by a black dashed line. Percentage occupancies of selected interactions are computed based on the number of structures in the trajectory in which the hydrogen bond is present

Interactions of the terminal nucleobase pair (C112:G205) of Watson–Crick DNA with the WT polymerase seen in the MD‐derived trajectory are consistent with experimental findings.^28^, ^29^, ^30^ Three intermolecular interactions between the C112:G205 nucleobase pair and protein residues Arg‐573, Arg‐746, and Asp‐785 are highly populated in the simulation, two of which (Arg‐746/G205 and Asp‐785/C112) involve the sugar‐phosphate backbone (Figure S5a). The third interaction is a hydrogen bond between the Arg‐573 side chain and the newly added nucleobase C112 (in the Nth position at the 3′‐end of the primer) (Figure S5a, Table S4). In addition, the side chain of Arg‐587, which is located in the ***αJ/K*** helical region of the palm domain, forms three highly populated hydrogen bonds to C111, located in the [N‐1]th position, in the MD trajectory (Orientation 1 of Figure 5a). This finding is consistent with prior proposals that Arg‐587 correctly positions the template strand through interactions with the [N‐1]th nucleotide thereby allowing the incoming dNTP to bind appropriately for reaction with the 3'‐OH in the ternary complex prior to primer extension.^29^

The interaction of Arg‐587 with the [N‐1]th nucleotide in the binary complex is also correlated with the presence of a salt bridge between Arg‐596 and Glu‐832 in the simulation trajectory of the WT KlenTaq/Watson–Crick DNA binary complex (Figures 5a and S6a). Two “coupled” arrangements of the Arg‐587 and Arg‐596 side chains located at the ends of the ***αJ/K*** helical region are also seen during the MD trajectory (Figure S6a). In the first arrangement (Orientation 1), which is present in a majority of sampled structures, Arg‐587 interacts with the [N‐1]th nucleotide (C111) and Arg‐596 is positioned to interact with the Glu‐826 and Glu‐832 side chains (Figure 5). In the second arrangement (Orientation 2) Arg‐587 interacts with the Nth nucleotide (C112) and Arg‐596 reorients to interact with the ***αH*** helix in the thumb domain (Figure 5).

These interactions at the growing end of the DNA duplex are altered in the MD simulations of the WT KlenTaq/AEGIS DNA binary complex (Table S5). For example, only two interactions with the Nth nucleotide (**Z**112) are observed, in which Arg‐573 and Asp‐785 continue to form populated interactions to the nucleobase and sugar‐phosphate backbone of **Z**112 (Figure S5b). Moreover, the AEGIS nucleobase **P**205, located in the template strand, becomes hydrogen bonded to the Gln‐754 side chain, a protein/DNA interaction that is absent in the trajectory for the WT KlenTaq/Watson–Crick DNA binary complex (Figure S5a). Importantly, because of its functional role in primer extension, the Arg‐587 side chain interacts with **Z**112 rather adopting its optimal location in which it can interact with C111, the [N‐1]th nucleotide (Figure 5, Table S5). Arg‐596 is also oriented so as to interact with Asp‐452 in the ***αH*** helix at the base of the thumb domain presumably as a consequence of the altered conformational preference of the Arg‐587 side chain (Figure 5b). Finally, the salt bridge between Arg‐596 and Glu‐832 is absent in the MD trajectory computed for the WT KlenTaq/AEGIS DNA binary complex.

### 2.5 Both C:G and Z:P are accommodated in the active site of the KlenTaq variant

In contrast to what is observed for the WT polymerase, the Nth nucleotide pair in both binary complexes of the KlenTaq variant is stabilized by interactions between the phosphate backbone and Arg‐746 and Arg‐785 (Figure S5b, Tables S6, S7). A direct interaction between G206 and both Gln‐754, and Arg‐573 is also present in the KlenTaq variant/Watson–Crick DNA binary complex. Arg‐573 is also seen to interact with C207. When AEGIS DNA is bound by the KlenTaq variant, however, the Gln‐754/**P**206 interaction is maintained but Arg‐573 binds to **Z**112 (Table S6, S7).

Importantly, and unlike what is observed for the WT polymerase, Arg‐587 preferentially binds to the [N‐1]th nucleotide in the primer (C111 or **Z**112) in *both* binary complexes involving the KlenTaq variant (Figure S7a, Table S6, S7). This finding suggests that the salt bridge between the side chains of Arg‐596 and Glu‐832 in the KlenTaq variant is not necessarily required to maintain the Arg‐587/[N‐1]th nucleobase interaction (Figure S7b).

**Figure 6:**
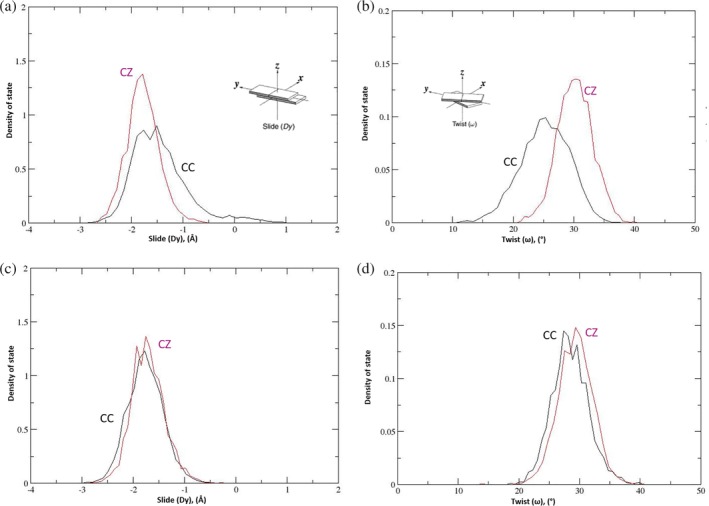
Representative histograms showing the distribution of slide and twist values for the dinucleotide step involving the (N‐1)th and Nth nucleobase pairs throughout the MD trajectories. (a) Slide and (b) twist values in the MD simulations of the WT polymerase binary complexes. (c) Slide and (d) twist values in the MD simulations of the variant polymerase binary complexes. In all Figures CC and C**Z** refer to C111/C112 and C111/**Z**112 in the Watson–Crick and AEGIS DNA primer, respectively. The insets showing the molecular definitions of slide and twist for dinucleotide base pairs are taken from Lu and Olson^31^

Although the DNA duplexes maintain their helical structure and bending angle in all MD simulations, the distribution of the slide and twist angles^31^ in the CC dinucleotide step in the Watson–Crick primer (slide: −1.3 and − 1.9 Å, twist: 25°) differ from those of the cognate (C**Z**) step in the AEGIS primer (slide: −1.9 Å, twist: 30°) (Figure 6a,b). This finding again supports the idea that WT KlenTaq binds DNA differently when the **Z**:**P** nucleobase pair replaces C:G whereas both Watson–Crick and AEGIS DNA are bound in a similar manner by the KlenTaq [variant Figure 6c,d]. The molecular origin of these differences in the slide and twist angle distributions are difficult to assign to specific protein/DNA interactions or individual amino acid substitutions in the Klentaq variant, although they may be associated with the altered electrostatic and dispersion properties of the **Z**:**P** nucleobase pair.^13^ As far as we can tell from our MD trajectories of the four binary complexes, the 3'‐OH in either of the terminal C or **Z** nucleotides adopts a similar location and participates in the same hydrogen bonding interactions (Figure S8). We cannot rule out the possibility, however, that longer MD simulations might exhibit altered positioning of the 3'‐OH in the Watson–Crick and/or AEGIS binary complexes.

### 2.6 M444 V and D551E substitutions affect the dynamics and DNA binding of the evolved KlenTaq variant

**Figure 7:**
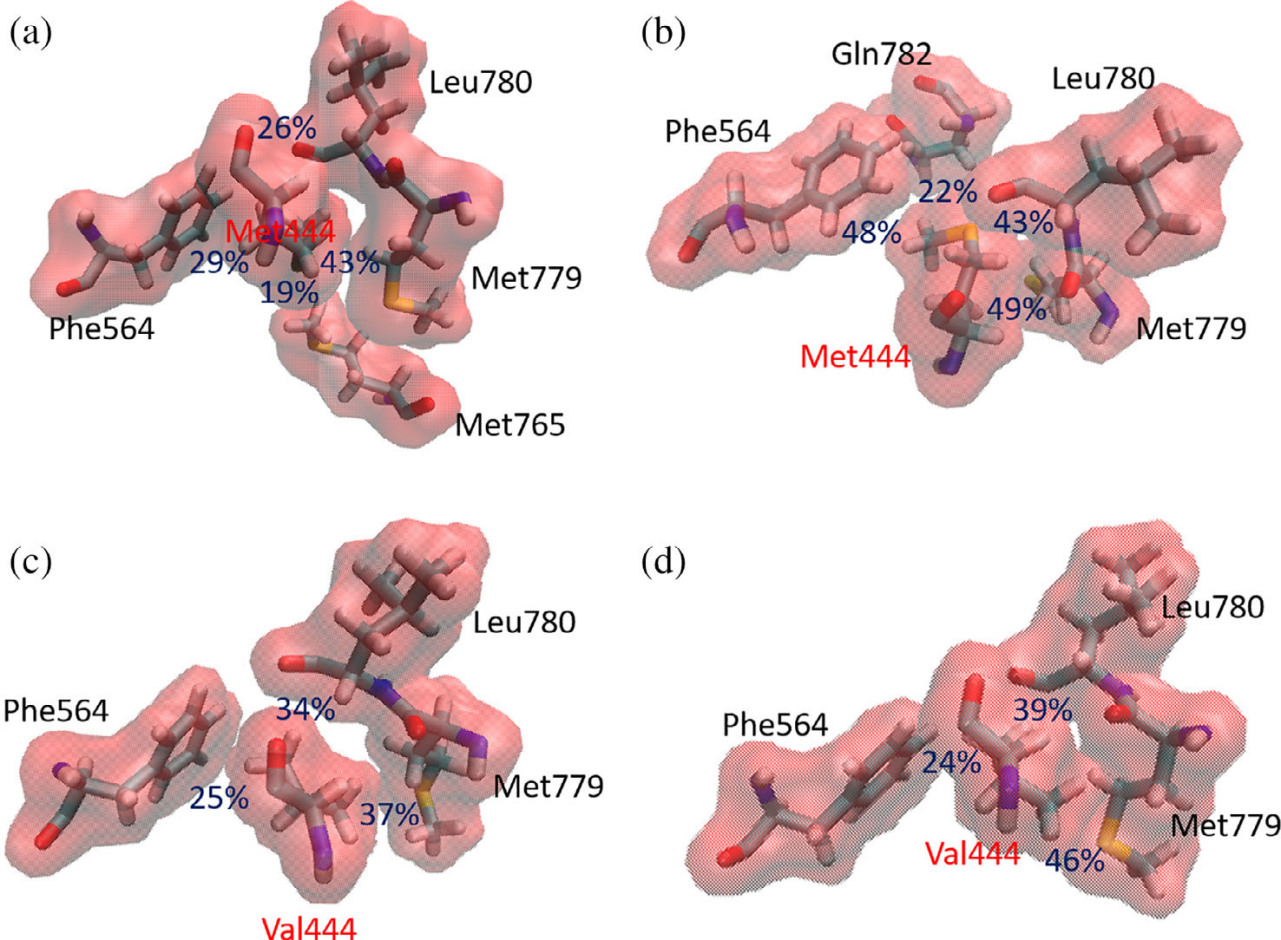
Interactions between residue 444 and surrounding residues in the MD trajectories of (a) the WT polymerase/Watson–Crick DNA binary complex, (b) the WT polymerase/AEGIS DNA binary complex, (c) the variant polymerase/Watson–Crick DNA binary complex, and (d) the variant polymerase/AEGIS DNA binary complex. The percentage occurrence (blue) of a specific interaction throughout the MD trajectory is also shown

Throughout the MD trajectory of the WT KlenTaq/Watson–Crick DNA binary complex, Met‐444 is held within a hydrophobic pocket defined by the side chains of residues Phe‐564 and Met‐779, which are located in the ***β6*** and ***β12*** strands, respectively (Figures 7 and S9a). The ***β6*** strand participates in an anti‐parallel sheet linking the ***αI*** helix of the thumb with the ***αJ/K*** region, and ***β12*** in the palm domain is directly connected to the ***αQ*** helix in the fingers. When WT KlenTaq is bound to Watson–Crick DNA, the Met‐444 side chain makes an additional interaction with Met‐765 that can be replaced by Gln‐782, when the wild‐type polymerase is bound to AEGIS DNA (Figure 7a,b). The introduction of valine in this position (Val‐444), however, means that the smaller side chain only forms populated interactions with Phe‐564, Met‐779 and Leu‐780 in the simulations of both KlenTaq variant/Watson–Crick and KlenTaq variant/AEGIS DNA binary complexes (Figure 7c,d). The consequence of this reduced set of interactions is that the ***αG*** helix becomes more flexible in the variant compared to the WT polymerase (Figure S9b). Our calculated result is again in agreement with inferences about the elasticity of the ***αG*** helix based on the X‐ray crystal structure of the KlenTaq variant/AEGIS DNA binary complex.^24^ Replacing Asp‐551 (located in the ***αI*** helix close to the ***αG*** helix) by glutamate also impacts the flexibility of the ***αG*** helix based on the MD simulations of the four binary complexes. Thus, the presence of the longer side chain at residue 551 permits a shorter (and stronger) salt bridge to the Arg‐547 side chain located on the ***αH*** helix than is present in WT KlenTaq (Figure S10b). When AEGIS DNA is complexed within the KlenTaq variant, the interaction between Asp‐551 and Arg‐547 has the effect of stiffening the thumb thereby moving the ***αG*** helix toward the fingers and giving rise to an anti‐correlated motion of the ***αG***, ***αP***, ***αQ***, and ***αR*** helices with the fingers domain (green and orange boxes in Figure 4 [right/bottom]).

### 2.7 Single amino acid substitutions fail to capture the properties of the evolved polymerase‐DNA complexes

**Figure 8:**
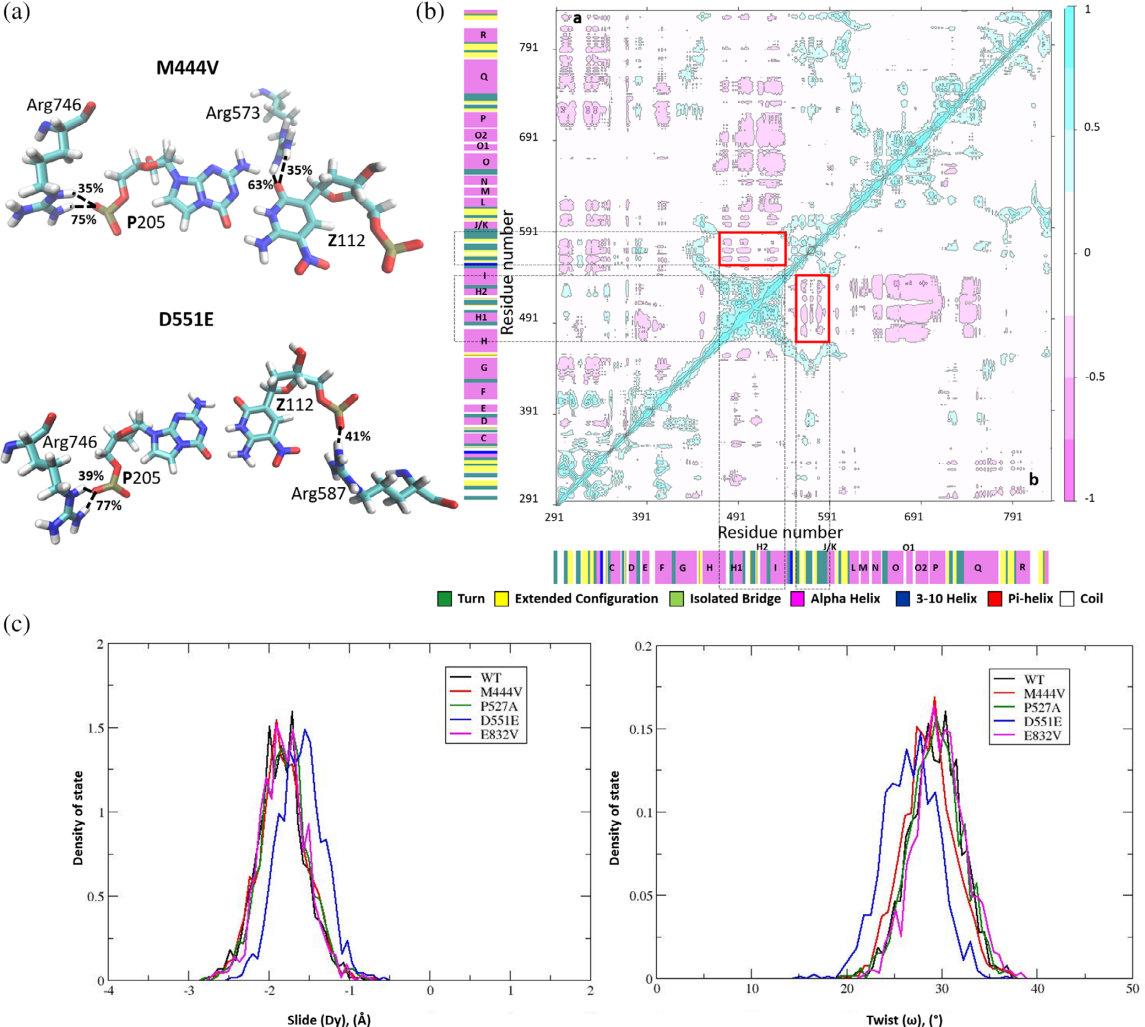
(a) Hydrogen bonds (dashed lines) between the **P**:**Z** nucleobase pair and protein residues, which are seen in the trajectories with greater than 30% occupancy in the MD trajectories computed for the M444 V KlenTaq variant/AEGIS DNA (top) and D551E KlenTaq variant/AEGIS DNA (bottom) binary complexes. Percentage occupancies of selected interactions are computed based on the number of structures in the trajectory in which the hydrogen bond is present. Atoms in named residues are colored according to the scheme: C, cyan; H, white; N, blue; O, red; P, orange. (b) Dynamical cross correlation maps computed for the M444 V KlenTaq variant/AEGIS DNA (top triangle) and D551E KlenTaq variant/AEGIS DNA (bottom triangle) binary complexes. Correlated (range: 0.25 to 1) and anti‐correlated (range: −0.25 to −1) motions are colored from light to dark blue and pink, respectively. Areas rendered in white correspond to non‐correlated motions (range: −0.25 to 0.25). Secondary structural elements are also included on the map. See text for details. (c) Slide (left) and twist (right) values for the C111/**Z**112 dinucleotide step of the AEGIS DNA primer in the MD simulations of the binary complexes of AEGIS DNA bound to WT KlenTaq and the four single‐point (M444 V, P527A, D551E, and E832V) KlenTaq variants

Additional MD simulations (only one repeat per variant) of the binary complexes for four single‐point KlenTaq variants were performed to disentangle the contributions of the individual residue replacements to altering the properties of the evolved enzyme. All of these binary complexes were stable under our simulation conditions (Figure S11) and the resulting trajectories were compared to that obtained for the WT KlenTaq/AEGIS DNA binary complex. In particular, we wanted to ascertain which, if any, of the residue substitutions corrected mispositioning of arginine side chains about the **P**:**Z** nucleobase pair in the AEGIS DNA duplex. No change was observed for the polymerase/**Z**112 interactions in the simulations of the P527A and E832V KlenTaq variants compared to those discussed above for the WT polymerase/AEGIS DNA complex (Figure S12). This was not the case, however, in the MD simulation of the M444 V variant/AEGIS DNA binary complex in which only the Arg‐573 side chain interacted with **Z**112 (Figure 8a). Altered polymerase/**Z**112 interactions were also observed for the D551E variant/AEGIS DNA binary complex in which only the Arg‐587 side chain can interact with the nonnatural nucleotide, albeit via an electrostatic interaction with the phosphate group in the oligonucleotide backbone (Figure 8a). As importantly, the motions of the M444 V KlenTaq variant do permit the side chain of Arg‐587 to hydrogen bond to C111, the [N‐1]th nucleotide (70–73% occurrence). In addition, the DCCMs for the M444 V and D551E KlenTaq variants (Figure 8b) reveal an anti‐correlated motion between the palm and thumb domains, which is absent in the P527A and E832V KlenTaq variants (Figure S13b). Turning the effects of individual residue substitutions on the structural properties of the template/primer duplex about the nonnatural **P**:**Z** nucleobase pair in the AEGIS DNA, we find that the C**Z** dinucleotide slide and twist distributions observed for the M444 V, P527A, and E832V KlenTaq variants are essentially identical to those seen for the WT polymerase. On the other hand, substituting Asp‐551 by Glu results in the C**Z** dinucleotide slide and twist distributions becoming more like that observed for the CC dinucleotide when Watson–Crick DNA is bound by the WT polymerase (Figures 6a,b and 8c). In addition, we note that the interaction of Asp‐785 with **Z**112 is absent in both MD trajectories computed for the M444 V/AEGIS DNA and D551E/AEGIS DNA complexes, suggesting that the addition of d**Z**TP to the primer will be slowed in both of these KlenTaq single substitution variants.

## 3 CONCLUSIONS

These MD simulations systematically explore the role of dynamical motions within the WT and evolved KlenTaq polymerases involved in positioning of Watson–Crick or AEGIS DNA template/primer substrates. In the absence of substrate, the amino acid substitutions within the evolved polymerase have no effect on the overall dynamical properties of the enzyme. In the presence of either Watson–Crick or AEGIS template/primer, however, the dynamical motions of the evolved KlenTaq are distinct from those of the WT polymerase. Specifically, WT KlenTaq is unable to maintain sets of correlated motions and specific interactions of amino acid residues required to appropriately position **P:Z** in the active site. The four amino acid substitutions in the KlenTaq variant resolve this problem by altering the flexibility of key segments of the enzyme with the consequence that the enzyme can bind to Watson–Crick and AEGIS DNA in an equivalent fashion.

Our MD simulations also confirm the proposal^24^ that the KlenTaq variant has increased flexibility compared with the WT polymerase, which facilitates positioning of the Arg‐587 side chain to interact with the [N‐1]th nucleotide in the growing strand of both Watson–Crick and AEGIS DNA duplexes. Replacing Met‐444 in the ***αG*** helix and Asp‐551 at the base of the thumb domain by valine and glutamate, respectively, allows the fingers, palm and thumb domains in the KlenTaq variant to bind AEGIS DNA in an optimal conformation. All three mutations are synergistic. Changing Met‐444 to valine increases the flexibility of the ***αG*** helix thereby ensuring an optimal interaction of Arg‐587 with both Watson–Crick and AEGIS DNA duplexes. Replacing Asp‐551 by glutamate rigidifies the base of the thumb and also increases the flexibility of the ***αG*** helix. Our calculations also confirm that replacing Pro‐527 by alanine permits the tip of the thumb to be more flexible,^24^ allowing the variant to bind both Watson–Crick and AEGIS DNA and facilitating the interaction of Asp‐785 with the newly added nucleotide (C112 or **Z**112) in the binary complex. In complexes of WT KlenTaq, salt bridge formation between Glu‐832 and Arg‐596 is correlated with positioning of the Arg‐587 side chain. This is no longer possible for the KlenTaq variant because Glu‐832 is substituted by valine. No obvious dynamical changes were observed, however, for the E832V KlenTaq variant.

Both MD simulation and X‐ray crystallography indicate that increasing domain flexibility will be required for polymerases capable of replicating DNA containing nonnatural nucleobases with high efficiency. This appears to be the case even for DNA duplexes that look very similar to those containing only Watson–Crick nucleobase pairs. In identifying correlated residue/residue and residue/nucleotide interaction networks, hinge regions, and domain movements required for productive positioning of both natural and unnatural template/primer substrates in the active site of the KlenTaq variant, our calculations lay a foundation for screening altered KlenTaq polymerases with potential use in not only replicating hachimoji DNA but also the shape complementary nucleobases developed by Romesberg^2^ and Hirao.^7^ Further, our comparative analyses of MD simulations for all possible combinations of WT and evolved polymerases complexed to Watson–Crick and AEGIS DNA provides a general, structure‐based strategy for obtaining polymerases capable of replicating expanded genetic alphabets.

## 4 METHODS

### 4.1 Parameterization of the nonnatural nucleobases

N9‐methylated forms of the **Z** and **P** nucleobases were geometry optimized at the HF/6‐31G* level of theory using the NWChem software package^32^ prior to obtaining RESP charges^33^ subject to the constraint of zero net charge. This procedure is consistent with the GAFF2 parameterization of small molecules.^34^ Partial charges on the sugar‐phosphate atoms in the corresponding nucleotide fragments were taken from the PARMBSC1 force field used to model nucleic acids,^35^ with the desired overall charge being obtained by adjusting the initial partial charges on C1' and H1' (Table S2).^36^ Force field parameters for the bonding interactions were obtained from PARMBSC1 and GAFF2. The resulting set of parameters for the two nonnatural nucleobases are provided elsewhere (Data S1).

### 4.2 Building the initial models of WT KlenTaq, the evolved KlenTaq variant and the binary complexes

An initial model of the evolved KlenTaq polymerase variant bound to template (5'‐AAAG**P**GCGCCGTGGTC‐3′) and primer (5'‐GACCACGGCGC**Z**‐3′) DNA (Figure S1b) was prepared from the corresponding X‐ray crystal structure (PDB: http://firstglance.jmol.org/fg.htm?mol=5W6Q).^24^ Unobserved residues (291–293, 642–665, and 832) in the original X‐ray crystal structure of the evolved KlenTaq variant were added based on their positions in other structures of WT KlenTaq (PDB:http://firstglance.jmol.org/fg.htm?mol=4KTQ 30 and PDB:http://firstglance.jmol.org/fg.htm?mol=1BGX 37). Energy minimization was performed to remove steric clashes in the initial model using the ff14SB^38^ and PARMBSC1^35^ force field parameters for the protein and DNA components, respectively, and our “in‐house” parameters for the **Z**:**P** nucleobase pair. These calculations together with subsequent equilibration and MD simulations employed the PMEMD module implemented in the Amber2016 software suite.^39^, ^40^ The resulting model of the KlenTaq variant/AEGIS DNA binary complex was then used to construct models of the apo‐forms of WT KlenTaq and the evolved KlenTaq variant, and the KlenTaq variant/Watson–Crick, the WT KlenTaq/Watson–Crick and the WT KlenTaq/AEGIS DNA binary complexes. Similarly, the last frame of WT KlenTaq/AEGIS DNA binary complex was used to construct the initial models of the binary complexes in which AEGIS DNA was bound to the four single‐point KlenTaq variants. Each of these models was placed in a box of explicit TIP3P^41^ water molecules containing K^+^ and Cl^−^ ions to yield an ionic strength of 10 mM (Table S3). These counterions were chosen in order to mimic the conditions under which the KlenTaq variant was obtained by directed evolution.^23^ Each system was energy minimized to remove bad contacts, heated, and then equilibrated for 100 ns to allow the quasi‐immobile ions sufficient time to equilibrate prior to the production phase.^42^ Three independent MD simulations of 110 ns were then performed on each of the six systems in the NPT ensemble (T = 323 K and *p* = 100,000 Pa). Models of the binary complexes of the M444 V, P527A, D551E, and E832V single‐point KlenTaq variants bound to AEGIS DNA were built from the last frame of the WT KlenTaq/**Z**:**P**‐containing DNA complex. These model structures were then solvated and equilibrated following the procedures outlined above, and MD trajectories (110 ns) determined using identical simulation conditions to those outlined above. Periodic boundary conditions were used in all MD simulations, with an 8 Å cutoff being used for non‐bonded interactions, and particle‐mesh Ewald methods were used to describe long‐range electrostatics.^43^, ^44^, ^45^ The temperature and pressure of each system was maintained using Langevin constraints,^46^ and the SHAKE algorithm was used to constrain bonds involving hydrogen atoms,^47^ which allowed the use of 2.0 fs time steps.

### 4.3 Analysis of the MD trajectories

Structures were sampled at 100 ps intervals in each of the 23 MD trajectories to give a combined 3,300 “snapshots” for each of the six WT and variant Klentaq systems, and 1,100 “snapshots” for the four single variants. These trajectories that were analyzed using the DynDom,^26^ 3DNA,^31^ Bio3D,^27^ CPPTRAJ,^48^ and VMD^49^ software packages. Thus, DCCMs were computed based on the motions of the C_α_ carbons of the protein using algorithms implemented in the Bio3D library in “R”.^27^ These maps reveal the extent to which the motions of two C_α_ carbons are correlated during the MD trajectory, with the correlation coefficient for the residue pair varying over a range of −1 to +1. When the C_α_ carbons have completely correlated motions (identical phase space, period and direction), the correlation coefficient has a value of +1. For “anti‐correlated” motions, in which the correlated motions take place in opposite directions, the value of the correlation coefficient is −1. For completely uncorrelated motions, the correlation coefficient has a value of zero. The Bio3D library was also used to perform PCA for all snapshots obtained in the combined MD trajectories for each of the six systems.

Reference structures needed for the DynDom analysis were obtained by averaging the coordinates of the last snapshot in each of the three independent MD simulations for WT Klentaq, the Klentaq variant and four binary complexes (WT KlenTaq/Watson–Crick DNA; WT KlenTaq/AEGIS DNA; KlenTaq variant/Watson–Crick DNA; KlenTaq variant/AEGIS DNA). The domain motions of each reference structure compared to the X‐ray crystal structure of WT KlenTaq in a binary complex (PDB: http://firstglance.jmol.org/fg.htm?mol=3SZ2)^30^ were then obtained using the DynDom package^26^ by aligning the C_α_ carbons of the two proteins. Helical parameters for the DNA molecules in each of the four complexes throughout the MD trajectories were calculated using the 3DNA^31^ and do_x3DNA programs.^50^ Hydrogen bonds were identified in the simulations assuming that the heavy‐atom donor (D)/acceptor (A) distance cutoff was 3.0 Å and that the D‐H‐A angle was in the range of 135–180°. Populated hydrogen bonds were defined to be present in at least 10% of the sampled structures in each MD trajectory except for those between the Arg‐587 side chain and the nitro group of the **Z** nucleobase

## Supporting information


**Appendix S1**: Supporting information
