# Supplementary material for: Building better enzymes: Molecular basis of improved non‐natural nucleobase incorporation by an evolved DNA polymerase
Source: Protein Sci. 2019 Nov 14;29(2):455–68. doi: 10.1002/pro.3762 (PMC6954703; doi:10.1002/pro.3762)
Supplement: Supplementary file 1 — Appendix S1: Supporting information [file PRO-29-455-s001.pdf]

SUPPLEMENTARY INFORMATION

Building Better Enzymes: Molecular Basis of Improved Non-Natural Nucleobase Incorporation by an Evolved DNA Polymerase

Zahra Ouaray[1], Isha Singh[2,3], Millie M. Georgiadis[2] and Nigel G. J. Richards[1,*]

[1] School of Chemistry, Cardiff University, Cardiff, CF10 3AT, United Kingdom
[2] Department of Biochemistry & Molecular Biology, Indiana University School of Medicine, Indianapolis, IN 46202, United States of America
[3] Present address: Department of Pharmaceutical Chemistry, University of California, San Francisco, San Francisco, CA 94143, United States of America

*Correspondence: Nigel G. J. Richards, School of Chemistry, Cardiff University, Park Place, Cardiff, CF10 3AT, United Kingdom. E-mail: RichardsN14@cardiff.ac.uk.

Supplementary Results and Discussion

KlenTaq and the evolved KlenTaq variant have similar dynamical properties in the absence of DNA.

MD simulations of wild type (WT) KlenTaq polymerase and the evolved KlenTaq variant in water show that there is little difference in the dynamics of the two enzymes in the absence of bound DNA. Both apo-enzymes maintain their structures throughout the simulations [Fig. S2] and the residue-dependent RMS fluctuations show similar profiles with significant fluctuations being primarily located within the “thumb” and “fingers” domains [Fig. S3]. The similarity of motions in the “thumb”, “palm” and “finger” domains for both WT and evolved KlenTaq polymerases is evident from principal component analysis (PCA)[1] of the dynamical motions of the alpha carbons relative to those in the equilibrated structure of WT KlenTaq when bound to template and primer DNA containing only Watson-Crick nucleobase pairs. Thus, the most important components (PC1 and PC2), which capture 37% of the coordinate variance during the simulations, correspond to movement of the tip of the “thumb” domain towards (PC1), and away from (PC2), the “fingers” domain [Fig. S4(A)]. Projecting the simulation snapshots along PC1 and PC2 shows that both WT KlenTaq polymerase and the evolved KlenTaq variant explore the same phase space [Fig. S4(A)]. Independent confirmation of this conclusion is provided by DynDom[2] analysis [Fig. S4(B)], which shows that residues in the tip of the “thumb” domain move away from the “fingers” and down towards to the exonuclease domain. This motion is characterized by a rotation of 34.6° and translation of -1.7 Å in WT KlenTaq and a rotation of 29.8° and a translation of -1.5 Å for the evolved KlenTaq variant [Table S1], and the domain that moves is identical in both of the apo-polymerases [Fig. S4(B)]. Further support is provided by dynamic cross-correlation maps (DCCMs)[8] constructed from the two MD

trajectories showing that motions of residues in (i) the thumb and fingers, and (ii) the thumb and palm, domains are anti-correlated in both enzymes [Fig. S4(C)].

Supplementary References

1. Skjaerven L, Martinez A, Reuter N (2011) Principal component and normal mode analysis of proteins; a quantitative comparison using the GroEL subunit. Proteins: Struct Funct Bioinform 79:232–243.

2. Taylor D, Cawley G, Hayward S (2014) Quantitative method for the assignment of hinge and shear mechanism in protein domain movements. Bioinformatics 30:3189–3196.

3. Hoshika S, Leal NA, Kim M-H, Kim M-S, Karalkar NB, Kim H-I, Bates AM, Watkins NE Jr, Santa Lucia HA, Meyer AJ, Das Gupta S, Piccirilli JA, Ellington AD, Santa Lucia J Jr, Georgiadis MM, Benner SA (2019) Hachimoji DNA and RNA: A genetic system with eight building blocks. Science 363:884-887.

4. Morris SE, Feldman AW, Romesberg FE (2017) Synthetic biology parts for the storage of increased genetic information in cells. ACS Synth Biol 6:1834–1840.

5. Hirao I, Kimoto M, Yamashige R (2012) Natural versus artificial creation of base pairs in DNA: Origin of nucleobases from the perspectives of unnatural base pair studies. Acc Chem Res 45:2055–2065.

6. Malyshev DA, Romesberg FE (2015) The expanded genetic alphabet. Angew. Chem. Int. Ed. 54:11930-11944.

7. Roe DR, Cheatham TE (2013) PTRAJ and CPPTRAJ: Software for processing and analysis of molecular dynamics trajectory data. J Chem Theory Comput 9:3084-3095.

8. Case DA, Cheatham TE, Darden T, Gohlke H, Luo R, Merz KM, Onufriev A, Simmerling C, Wang B, Woods RJ (2005) The Amber biomolecular simulation programs. J Comput Chem 26:1668-1688.

9. Betz K, Malyshev DA, Lavergne T, Welte W, Diederichs K, Dwyer TJ, Ordoukhanian P, Romesberg FE, Marx A (2012) KlenTaq polymerase replicates unnatural base pairs by inducing a Watson-Crick geometry. Nat Chem Biol 8:612-614.

Table S1: DynDom domain motion analysis for apo-enzymes and the WT/Watson-Crick, WT/AEGIS, KlenTaq variant/Watson-Crick and KlenTaq variant/ AEGIS DNA binary complexes.

PDB file 1	PDB file 2	Fixed domain	Moving domain	Rotation angle (°)	Translation (Å)	% Closure	Bending residues
**ZP binary Chain A**	**3SZ2**	**306-453<br>458-461<br>542-579<br>594-597<br>600-824**	**454-457<br>462-541<br>580-593<br>598-599<br>825-828**	**3.8**	**-0.1**	**53.3**	**453-454<br>457-458<br>461-462<br>541-542<br>579-580<br>593-594<br>597-600<br>824-825**
WT no DNA	3SZ2	304 - 464<br>541 - 830	465 – 540	34.6	-1.7	64.9	463 - 468<br>538 – 547
Variant no DNA	3SZ2	306 - 464<br>544 – 829	465 – 543	29.8	-1.5	66.8	462 - 465<br>541 – 547
WT natural DNA	3SZ2	425 - 437<br>451 - 556<br>560 - 562<br>565 - 571<br>574 - 599<br>609 - 765<br>780 - 787<br>793 - 827	296 - 424<br>438 - 450<br>557 - 559<br>563 - 564<br>572 - 573<br>600 - 608<br>766 - 779<br>788 - 792	9.3	0.2	89.1	421 - 425<br>437 - 438<br>450 - 451<br>556 - 557<br>559 - 560<br>562 - 574<br>599 - 601<br>608 - 609<br>765 - 766<br>779 - 780<br>787 - 788<br>792 - 793
Variant natural DNA	3SZ2	296 - 310<br>312 - 317<br>322 - 333<br>341 - 354<br>368 - 368<br>370 - 626<br>668 - 672<br>704 - 814<br>818 - 830	311 - 311<br>318 - 321<br>334 - 340<br>355 - 367<br>369 - 369	7.2	0.1	92.4	310 - 312<br>317 - 318<br>320 - 322<br>327 - 334<br>340 - 341<br>354 - 355<br>367 - 370
			627 - 667<br>673 - 703<br>815 - 817	14.6	0.5	99.5	626 - 627<br>667 - 676<br>703 - 704<br>814 - 815<br>817 - 818
WT AEGIS	3SZ2	312 - 318<br>357 - 381<br>430 - 460<br>535 - 609<br>765 - 800<br>820 - 823	295 - 311<br>319 - 356<br>382 - 421	7.8	-0.2	12.3	311 - 312<br>318 - 319<br>356 - 358<br>381 - 382<br>421 - 430
			477 - 534	6.4	0.2	82.4	460 - 477<br>534 - 535
			610 - 763<br>801 - 819	7.5	0.2	42.1	609 - 610<br>763 - 765<br>800 - 801<br>819 - 820
Variant AEGIS	3SZ2	416 - 440<br>451 - 452<br>454 - 461<br>535 - 542<br>544 - 547<br>567 - 573<br>584 - 584<br>594 - 600<br>606 - 764<br>788 – 822<br>295 - 415<br>441 - 450<br>453 - 453	543 - 543<br>548 - 562<br>580 - 583<br>585 - 593<br>601 - 605<br>765 - 787<br>823 - 830	9.0	0.1	87.8	415 - 416<br>440 - 441<br>450 - 454<br>542 - 544<br>547 - 548<br>562 - 567<br>573 - 580<br>583 - 585<br>593 - 594<br>600 - 601<br>605 - 606<br>764 - 765<br>787 - 788<br>822 - 823
			468 - 534	11.0	-2.4	57.5	461 - 468<br>534 - 535

Table S2: Atom types and partial charges for the **Z** and **P** nucleobases used in the MD simulations of AEGIS DNA-containing binary complexes. Atom types are taken from PARMBSC1 and GAFF2 force field. Charges for atoms in the sugar phosphate backbone were taken from PARMBSC1 and partial charges for the **Z** and **P** nucleobases were calculated as outlined elsewhere. The overall charge for each nucleotide was set to -1 by modifying the partial charges for C1' and H1'.

Z			P		
Atom name	Atom type	Charges	Atom name	Atom type	Charges
OP1	O2	-0.7761	O6	O	-0.68019
P	P	1.1659	C6	C	1.027419
OP2	O2	-0.7761	N1	NC	-0.95637
O5'	OS	-0.4954	C2	CA	1.146417
C5'	CI	-0.0069	N2	N2	-1.014116
H2	H1	0.0754	H15	H	0.42303
H3	H1	0.0754	H16	H	0.41416
C4'	CT	0.1629	N3	NC	-0.846649
H4	H1	0.1176	C4	CB	0.5044
C3'	CE	0.0713	N5	N*	-0.252421
H5	H1	0.0985	C7	CK	-0.18837
C2'	CT	-0.0854	H17	H5	0.228235
H7	HC	0.0718	C8	CK	-0.236506
H8	HC	0.0718	H18	H5	0.219649
O3'	OH	-0.6549	N9	N*	0.143379
H6	HO	0.4396	C1'	CT	0.081808
O4'	OS	-0.3691	H14	H2	0.107725
C1'	CT	0.002081	C2'	CT	-0.0854
H9	H2	0.104711	C3'	CE	0.0713
C1	CM	-0.149233	H11	H1	0.0985
C6	CM	0.107513	O3'	OS	-0.5232
H13	H4	0.149638	H12	HC	0.0718
C2	C	0.732751	H13	HC	0.0718
O2	O	-0.616152	O4'	OS	-0.3691
N3	N*	-0.6665	C4'	CT	0.1629
H10	H	0.394779	H10	H1	0.1176
C4	CA	0.809652	C5'	CI	-0.0069
N4	N2	-1.007104	H8	H1	0.0754
H11	H	0.432936	H9	H1	0.0754
H12	H	0.495312	O5'	OS	-0.4954
C5	CM	-0.567261	P	P	1.1659
N	no	1.021853	OP2	O2	-0.7761
ON2	o	-0.51215	OP1	O2	-0.7761
ON1	o	-0.611226			

Table S3: Water molecules and ions in the MD simulations of WT KlenTaq and the KlenTaq variant.

	Wild type			Mutant		
	**Natural DNA**	**Z/P**	**No DNA**	**Natural DNA**	**Z/P**	**No DNA**
**Number of ions**	35 K^+ (30 to neutralize system) and 5 Cl^-	35 K^+ (30 to neutralize system) and 5 Cl^-	12 K^+ (7 to neutralize system) and 5 Cl^-	34 K^+ (29 to neutralize system) and 5 Cl^-	34 K^+ (29 to neutralize system) and 5 Cl^-	11 K^+ (6 to neutralize system) and 5 Cl^-
**Water box dimension (Å) (Volume Å^3)**	89.3, 109.4, 95.9 (935807.7)	88.6, 106.7, 94.7 (895122.7)	93.8, 104.2, 94.7 (925594.1)	91.3, 104.6, 100.6 (960837.9)	89.3, 105.9, 95.4 (902857.6)	89.3, 107.3, 93.9 (899729.7)
**Number of atoms**	78192	74055	77884	79390	75370	75239

Table S4: Selected protein-DNA hydrogen bonds in the WT KlenTaq/Watson-Crick DNA binary complex. Only interactions that are populated to an extent of ≥ 30% are shown in the table.

	WT Watson-Crick DNA			
	**Acceptor**	**Donor**	**DonorH**	**Hydrogen bond (%)**
**Protein donor**	DG_108@OP2	SER_513@OG	HG	96
	DG_108@OP1	THR_514@OG1	HG1	93
	DG_108@OP1	THR_514@N	H	93
	DC_111@OP1	VAL_586@N	H	92
	DG_213@OP1	SER_486@OG	HG	89
	DG_107@OP1	THR_509@OG1	HG1	88
	DC_109@OP1	ARG_536@NH1	HH11	86
	DG_108@OP1	ARG_487@NH1	HH11	81
	DG_107@OP1	THR_509@N	H	81
	DG_205@OP2	ARG_746@NH2	HH21	80
	DC_109@OP2	SER_515@OG	HG	73
	DG_107@OP1	THR_506@OG1	HG1	65
	DG_205@OP2	ARG_746@NE	HE	58
	DC_109@OP2	ALA_516@N	H	58
	DC_209@OP1	ASP_578@N	H	53
	DG_208@OP1	SER_585@OG	HG	53
	DC_111@OP2	ARG_587@NE	HE	51
	DC3_112@O2	ARG_573@NH1	HH12	50
	DC_111@OP2	ARG_587@NH2	HH21	47
	DC_111@OP1	ARG_587@N	H	44
	DT_212@OP1	ASN_483@ND2	HD21	35
	DC3_112@O2	ARG_573@NH2	HH22	34
	DG_110@OP2	ARG_587@NH2	HH22	32
	DC_207@OP1	THR_569@OG1	HG1	31
	DT_212@OP1	ASN_485@ND2	HD22	30
	**Acceptor**	**Donor**	**DonorH**	**Hydrogen bond (%)**
**DNA donor**	ASN_583@OD1	DG_110@N2	H21	48
	ASP_785@OD1	DC3_112@O3'	HO3'	43
	ASP_785@OD2	DC3_112@O3'	HO3'	34

Table S5: Selected protein-DNA hydrogen bonds in the WT KlenTaq/AEGIS DNA binary complex. Only interactions that are populated to an extent of ≥ 30% are shown in the table.

	WT AEGIS DNA			
	**Acceptor**	**Donor**	**DonorH**	**Hydrogen bond (%)**
**Protein donor**	DG_108@OP2	SER_513@OG	HG	96
	DG_108@OP1	THR_514@N	H	94
	DC_111@OP1	VAL_586@N	H	92
	DG_108@OP1	THR_514@OG1	HG1	92
	DG_107@OP1	THR_509@OG1	HG1	89
	DC_109@OP1	ARG_536@NH1	HH11	87
	DG_208@OP1	SER_575@OG	HG	82
	DG_108@OP1	ARG_487@NH1	HH11	80
	DG_107@OP1	THR_509@N	H	79
	DG_213@OP1	SER_487@OG	HG	73
	DC_207@OP1	THR_569@OG1	HG1	68
	DC_109@OP2	ALA_516@N	H	68
	WZ_112@OP2	ARG_587@NH2	HH22	63
	DG_206@OP1	ARG_728@NH1	HH12	59
	DC_109@OP2	SER_515@OG	HG	58
	DG_107@OP1	THR_506@OG1	HG1	56
	WZ_112@O2	ARG_573@NH1	HH12	55
	WZ_112@OP2	ARG_587@NH1	HH12	52
	WZ_112@O2	ARG_573@NH2	HH22	46
	DT_212@OP1	ASN_483@ND2	HD21	43
	DC_111@OP2	ARG_587@NE	HE	43
	DC_111@OP1	ARG_587@N	H	40
	DC_111@OP2	ARG_587@NH2	HH21	34
	**Acceptor**	**Donor**	**DonorH**	**Hydrogen bond (%)**
**DNA donor**	SER_583@OG	DG_110@N2	H21	62
	ASP_785@OD2	WZ_112@O3'	H6	41
	GLN_754@OE1	WA_205@N2	H16	36

Table S6: Selected protein-DNA hydrogen bonds in the evolved variant/Watson-Crick DNA binary complex. Only interactions that are populated to an extent of ≥ 30% are shown in the table.

	Variant Watson-Crick DNA			
	**Acceptor**	**Donor**	**DonorH**	**Hydrogen bond (%)**
**Protein donor**	DG_108@OP2	SER_513@OG	HG	95
	DG_108@OP1	THR_514@N	H	93
	DG_108@OP1	THR_514@OG1	HG1	92
	DG_208@OP1	SER_575@OG	HG	92
	DC_207@OP1	THR_571@OG1	HG1	92
	DC_111@OP1	VAL_586@N	H	90
	DG_107@OP1	THR_509@OG1	HG1	89
	DC_109@OP1	ARG_436@NH1	HH11	86
	DG_206@OP1	ARG_728@NH1	HH12	85
	DG_108@OP1	ARG_487@NH1	HH11	79
	DG_107@OP1	THR_509@N	H	76
	DG_213@OP1	SER_486@OG	HG	74
	DC_109@OP2	ALA_516@N	H	67
	DG_205@OP1	ARG_746@NH2	HH21	64
	DC_109@OP2	SER_515@OG	HG	61
	DG_107@OP1	THR_506@OG1	HG1	60
	DC_209@OP1	SER_577@OG	HG	54
	DC_207@OP2	ALA_570@N	H	51
	DC_207@O5'	ARG_573@NH2	HH21	51
	DG_206@OP1	ARG_728@NH2	HH22	47
	DG_205@OP1	ARG_746@NE	HE	47
	DT_212@OP1	ASN_483@ND2	HD21	44
	DG_206@N3	ARG_573@NH2	HH22	44
	DC_209@OP1	ASP_578@N	H	44
	DG_110@N3	ASN_583@ND2	HD22	42
	DC_111@OP1	ARG_587@N	H	41
	DC_207@OP1	THR_571@N	H	38
	DC_111@OP2	ARG_587@NH2	HH21	34
	DC_111@OP2	ARG_587@NE	HE	34
	DG_110@OP2	ARG_587@NH2	HH22	30
	**Acceptor**	**Donor**	**DonorH**	**Hydrogen bond (%)**
**DNA donor**	GLN_754@OE1	DG_205@N2	H22	74
	ASP_785@OD1	DC3_112@O3'	HO3'	46

Table S7: Selected protein-DNA hydrogen bonds in the evolved variant /AEGIS DNA binary complex. Only interactions that are populated to an extent of ≥ 30% are shown in the table.

	Variant AEGIS DNA			
	**Acceptor**	**Donor**	**DonorH**	**Hydrogen bond (%)**
**Protein donor**	DG_108@OP2	SER_513@OG	HG	96
	DG_108@OP1	THR_514@N	H	94
	DG_108@OP1	THR_514@OG1	HG1	91
	DC_111@OP1	VAL_586@N	H	89
	DG_107@OP1	THR_509@OG1	HG1	86
	DC_109@OP1	ARG_536@NH1	HH11	85
	DG_208@OP1	SER_575@OG	HG	83
	DG_108@OP1	ARG_487@NH1	HH11	78
	DG_107@OP1	THR_509@N	H	77
	DG_213@OP1	SER_486@OG	HG	75
	DG_206@OP1	ARG_728@NH1	HH12	69
	DC_109@OP2	SER_515@OG	HG	66
	DC_207@OP1	THR_569@OG1	HG1	65
	WA_205@OP2	ARG_746@NH2	HH21	63
	DC_109@OP2	ALA_516@N	H	62
	WZ_112@O2	ARG_573@NH1	HH12	59
	DG_107@OP1	THR_506@OG1	HG1	59
	WA_205@OP2	ARG_746@NE	HE	47
	DT_212@OP1	ASN_483@ND2	HD21	46
	DC_111@OP2	ARG_587@NE	HE	46
	DC_111@OP1	ARG_587@N	H	43
	DC_111@OP2	ARG_587@NH2	HH21	42
	WZ_112@O2	ARG_573@NH2	HH22	42
	DC_209@OP1	SER_577@OG	HG	34
	**Acceptor**	**Donor**	**DonorH**	**Hydrogen bond (%)**
**DNA donor**	GLN_754@OE1	WA_205@N2	H16	46
	ASP_785@OD1	WZ_112@O3'	H6	41
	ASP_785@OD2	WZ_112@O3'	H6	34

Figure S1: (A) Unnatural nucleobase base pairs used in current artificially expanded genetic alphabets, which either interact via hydrogen bonding (AEGIS)[3] or by shape complementarity.[4-6] Hydrogen bonds are shown as dashed lines. (B) Sequences of the AEGIS DNA primer and template strands used in the MD simulations of the binary complexes. The corresponding Watson-Crick primer/template sequences were obtained by replacing **Z**112 and **P**205 (numbering used in PDB:5W6Q)[5] by C and G, respectively.

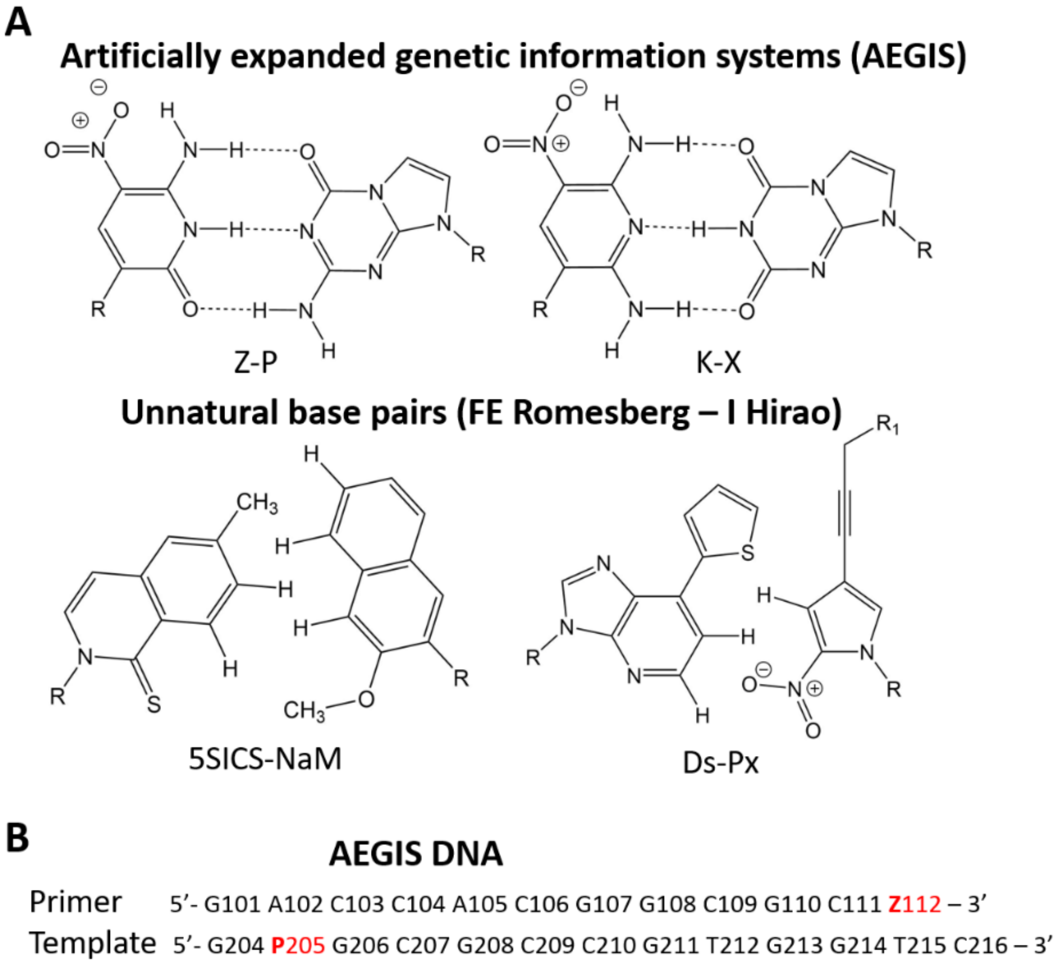

Figure S2: Root mean square deviation (RMSD) of the Cα positions for each simulated system relative to the equilibrated structure of the WT polymerase/Watson-Crick DNA binary complex, as calculated using CPPTRAJ[7] in the AMBER 2016 software suite.[8] An alignment of the reference structure (red) and the last frame of the MD simulation (blue) illustrate the stability of the system in the MD simulation.

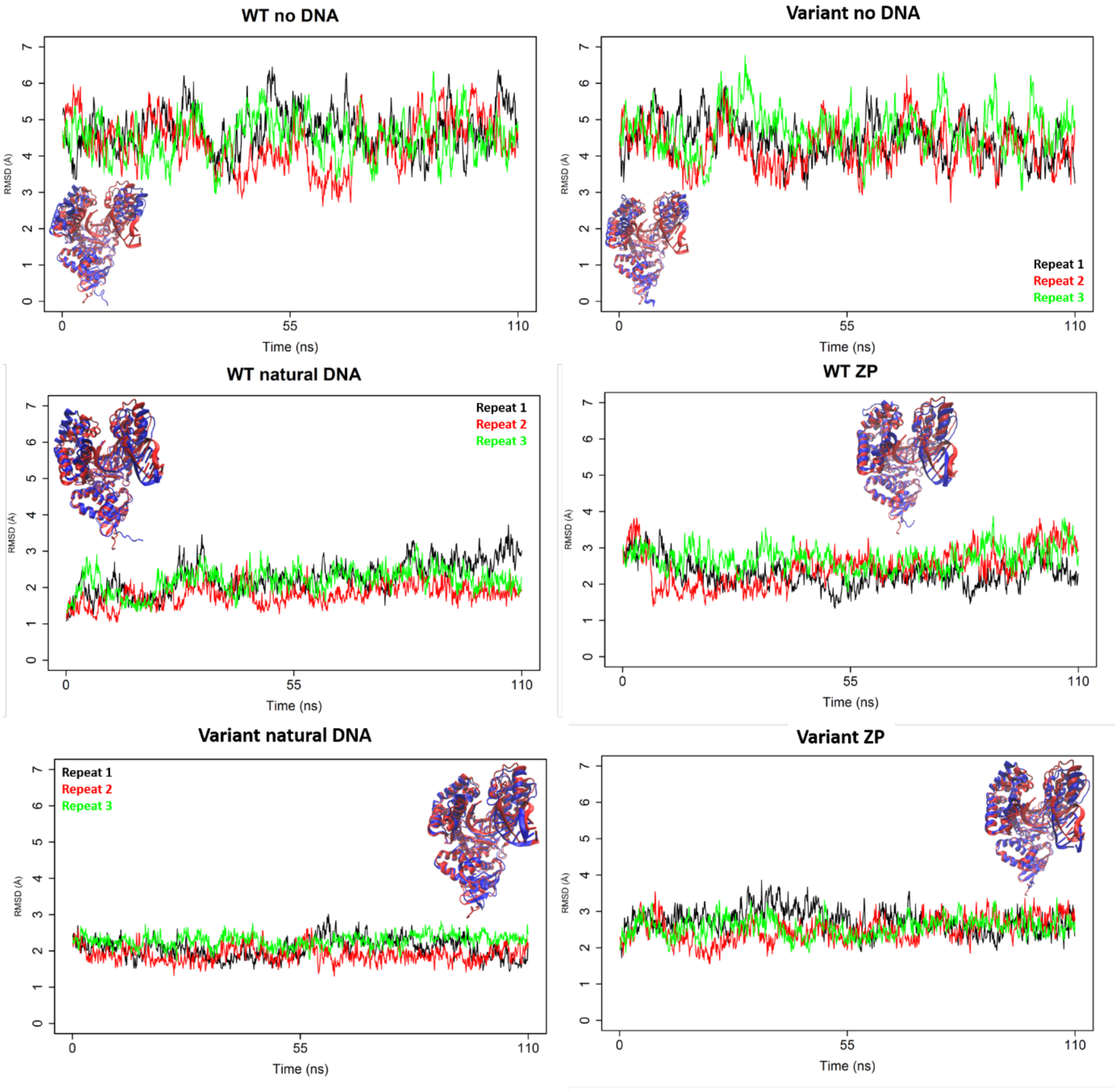

Figure S3: Average root mean square fluctuations (RMSF) calculated for residues in the MD trajectories of the two apo-enzymes and the four binary complexes. Residues located in the thumb and fingers domains are highlighted by the labelled bars.

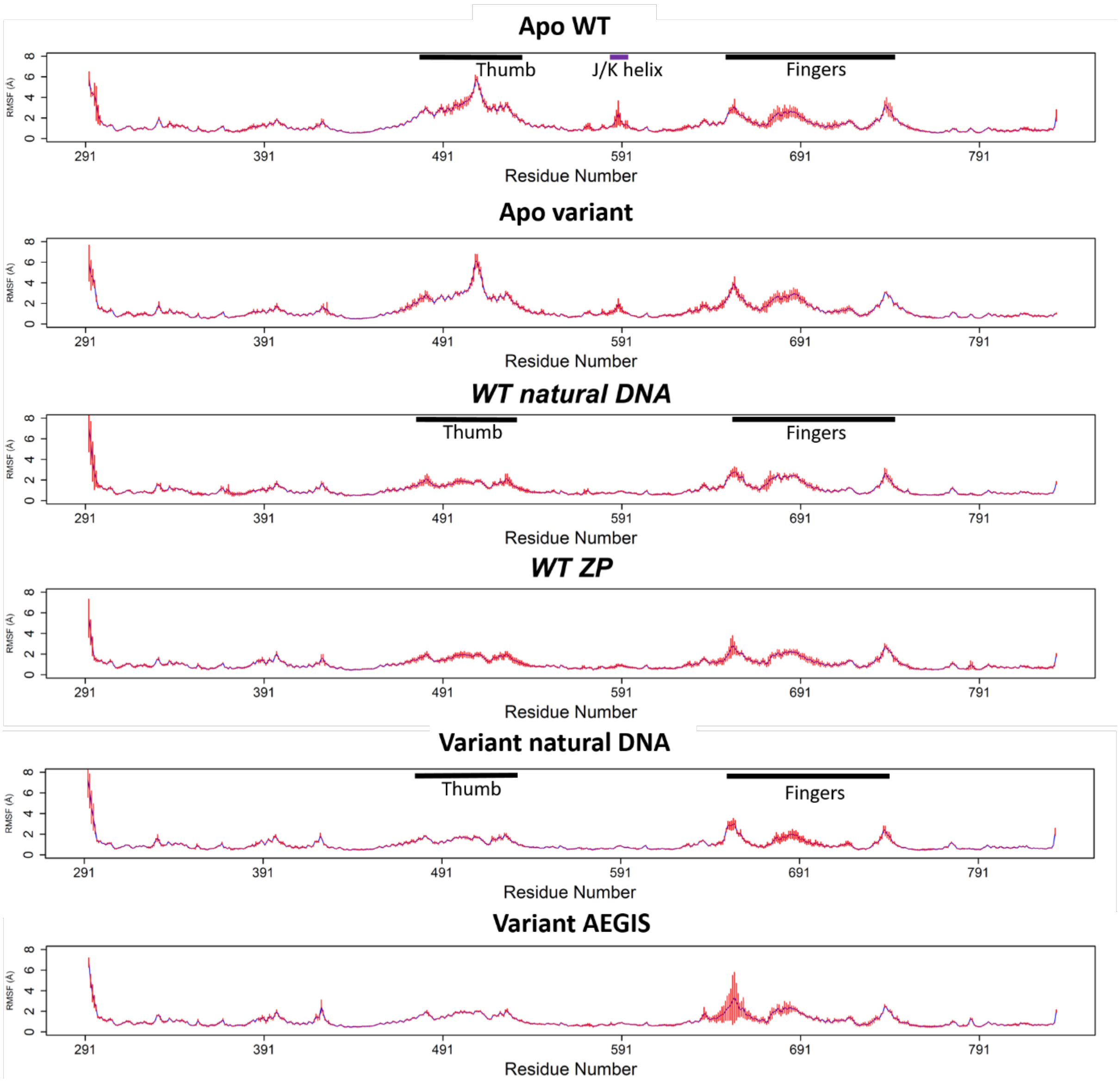

Figure S4: (A) Cartoon representation of the motions associated with the two principal components PC1 (left) and PC2 (center) describing the variance of the free WT and variant KlenTaq polymerases at early (red), intermediate (white) and late (blue) stages of the MD simulations. (Right) Projection of trajectory snapshots along PC1 and PC2 for free WT (blue) and variant (red) KlenTaq polymerases showing that the proteins explore the same regions of phase space. (B) C_α traces of the free WT (top) and variant (bottom) KlenTaq polymerases superimposed on that of the reference binary complex (grey) (PDB: 3SZ2).[9] The axes of domain motions are indicated by the arrows, the heads of which are colored to represent the moving domain (red). The fixed domain (blue) and regions that bend during the motion are also shown (green). (C) Dynamical cross correlation map (DCCM) of the free WT (a: top triangle) and variant (b: bottom triangle). Correlated (range: 0.25 to 1) and anti-correlated (range: -0.25 to -1) motions are colored from light to dark blue and pink, respectively. Areas rendered in white correspond to non-correlated motions (range: -0.25 to 0.25). Secondary structural elements are also included on the map

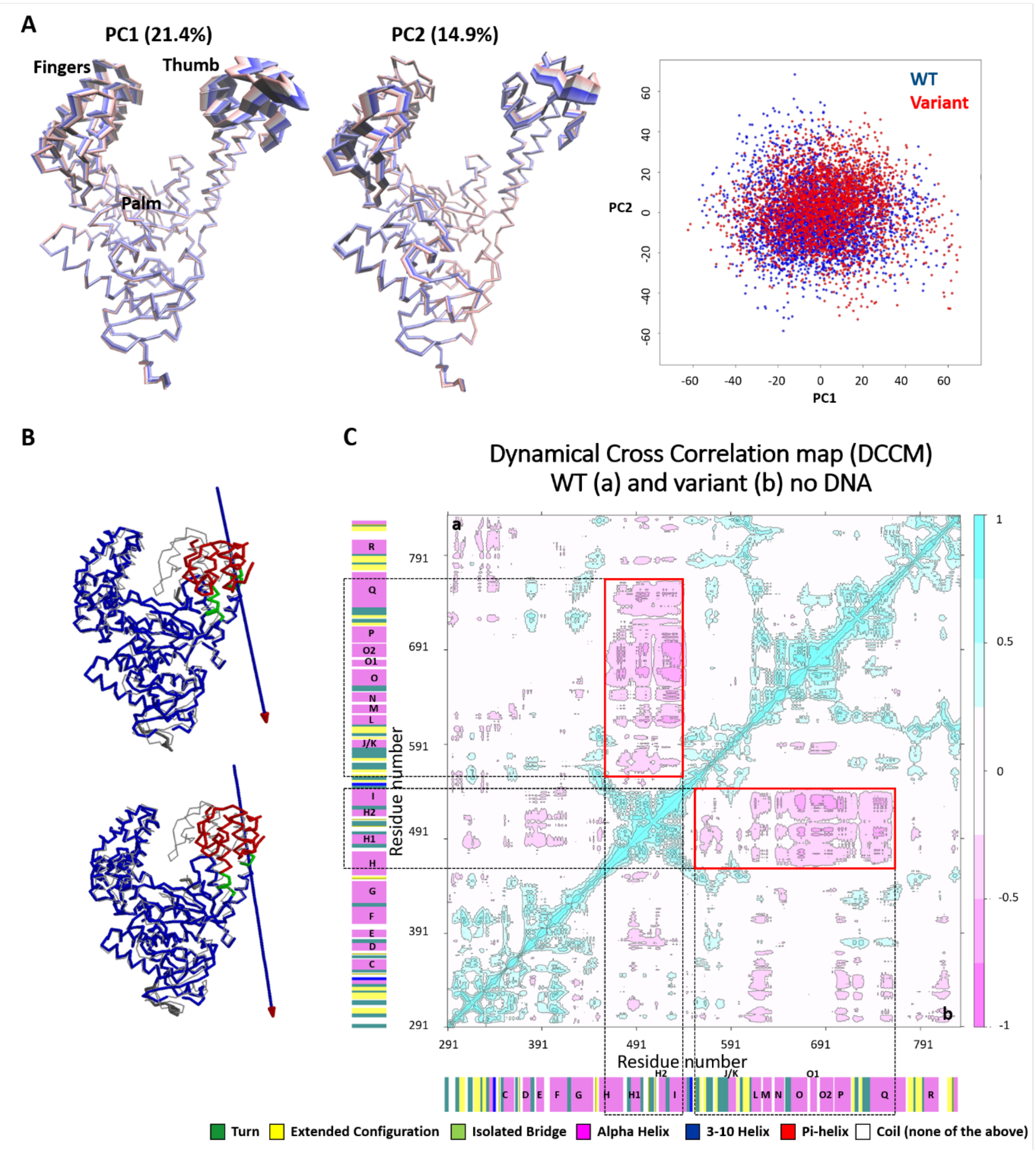

Figure S5: Hydrogen bond interactions between the enzyme and the terminal nucleobase pair for (A) WT KlenTaq bound to Watson-Crick (left) and AEGIS DNA (right), and (B) the evolved KlenTaq variant bound to Watson-Crick (left) and AEGIS DNA (right). For clarity the hydrogen bond donors and acceptors in DNA are shown in a different figure. The protein structure is in cartoon coloured in grey and the residues in liquorice and the atoms in named residues are colored according to the scheme: C, cyan; H, white; N, blue; O, red; S, yellow. The hydrogen bonds (≥ 30% occurrence) are represented by black dashed lines and their percentage of occurrence is shown in black.

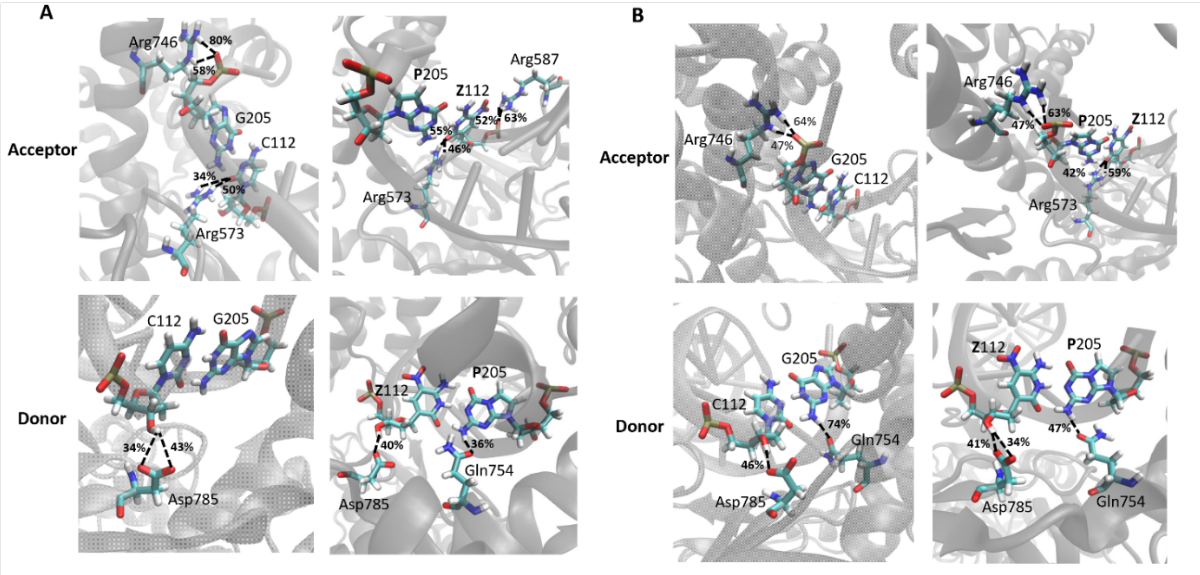

Figure S6: Cartoon showing orientations 1 and 2 of the ***αJ/K*** helix relative to residues Arg-587 and Arg-596. (A) Orientation 1 (left) positions Arg-587 toward the [N-1]th nucleotide and Arg-596 toward the Cter region. Orientation 2 (right) positions Arg-587 toward the newly added (Nth) nucleotide and Arg-596 toward the thumb domain. Purple boxes represent the ***αH*** and ***αJ/K*** helices and the Cter region is shown by the blue half-circle represents the Cter. Arg- 587 and Arg-596 are depicted by black lines and the [N-1]th and Nth nucleotide by boxes. (B) Orientations seen in the X-ray crystal structure of the evolved KlenTaq variant/AEGIS binary complex (5W6Q).[5] (Left/Middle) In chains C and G (orientation 1), Arg-596 is oriented towards the Cter region while Arg-587 binds to C111. (Right) In chain A (orientation 2) Arg-596 is oriented towards the thumb and Arg-587 binds to **Z**112. The ***αJ/K*** helix and the Cter region are colored by structure. C111 and **Z**112 nucleobases are drawn in liquorice and atoms in named residues are colored according to the scheme: C, cyan; H, white; N, blue; O, red. Side chains of Arg-587 and Arg-596 are coloured in blue and green in orientation 1 and orientation 2, respectively.

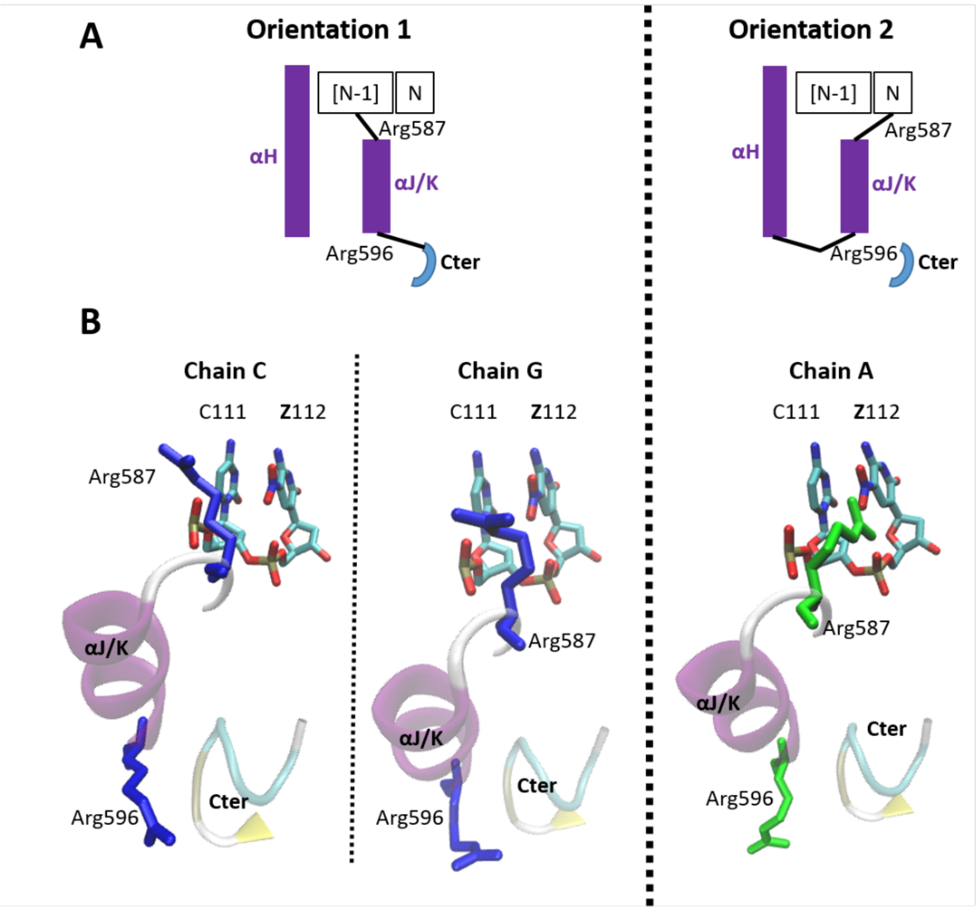

Figure S7: (A) Arg-587 hydrogen bond interactions with the [N-1]th nucleotide (orientation 1) and with the Nth nucleotide (orientation 2) in the (left) evolved KlenTaq variant/Watson-Crick DNA and (right) evolved KlenTaq variant/AEGIS DNA complexes. (B) Arg-596 hydrogen bond interactions with Glu-826 in the Cter region (orientation 1) and with Asp-452 in the ***αH*** helix of the thumb (orientation 2) in the (left) evolved KlenTaq variant/Watson-Crick DNA and (right) evolved KlenTaq variant/AEGIS DNA complexes. Cartoon representations show the ***αH*** and ***αJ/K*** helices and the Cter region. Atoms in named residues are colored according to the scheme: C, cyan; H, white; N, blue; O, red. Cα carbons are represented by blue spheres. The hydrogen bonds are represented by black dashed lines and their percentage of occurrence is shown in black.

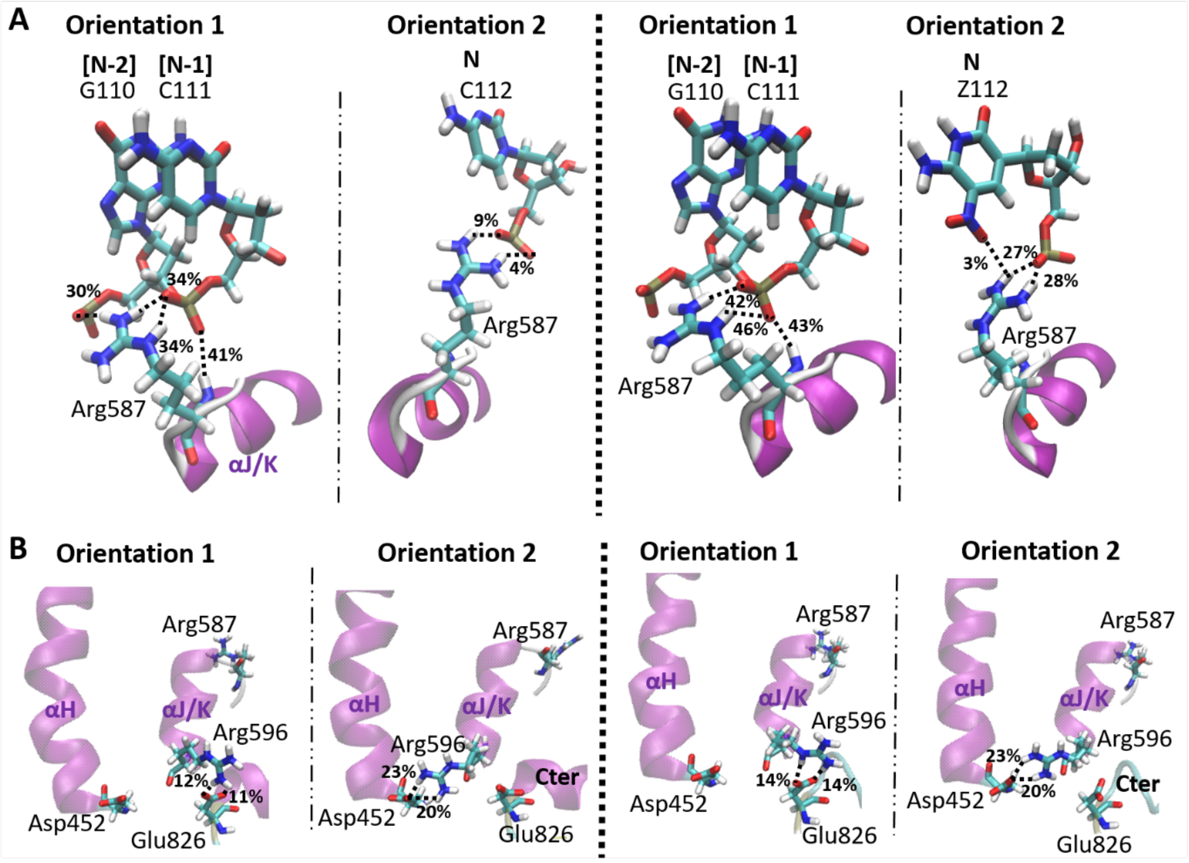

Figure S8: Position and hydrogen bonding interactions of the 3'-OH of C in the Watson-Crick (top) and **Z** in the AEGIS (bottom) DNA duplexes bound to the evolved Klentaq variant. Color: C, cyan; H, white; N, blue; O, red. Hydrogen bonds are indicated by dotted red lines.

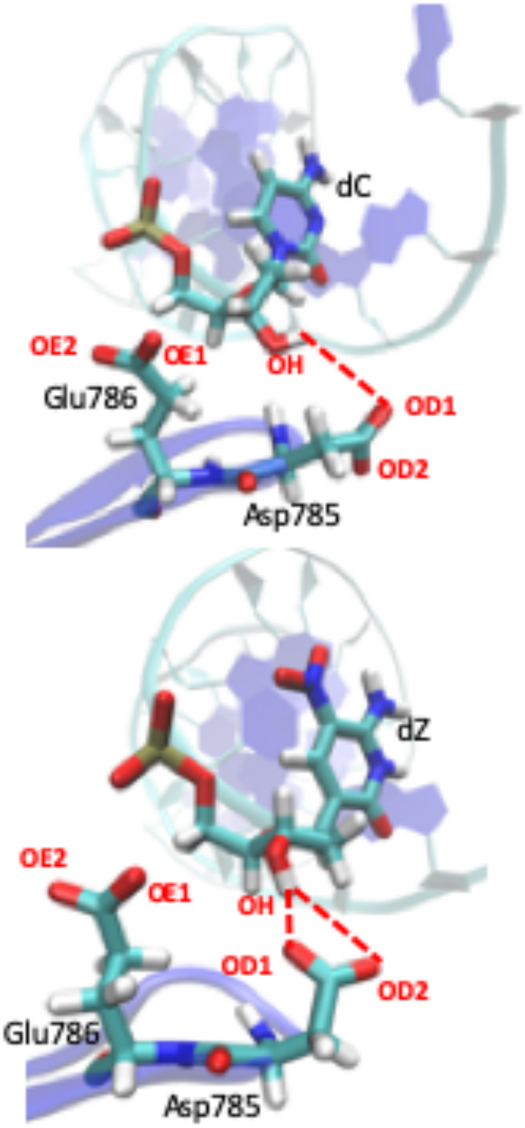

Figure S9: (A) Residues in WY KlenTaq close to Met-444. The protein is in coloured in grey and atoms in named residues are colored according to the scheme: C, cyan; H, white; N, blue; O, red; S, yellow. The ***αG*** helix is coloured in purple and strands ***β6*** and ***β12*** in red. (B) Histogram showing the variation in ***αG*** helix length during the MD trajectories based on the Cα-Cα distance between residues Glu-424 and Thr-447. The average distance is indicated by the blue line. Inset: Cartoon representation of the ***αG*** helix. Residues Glu-424 and Thr-447 are shown in liquorice and atoms are colored according to the scheme: C, cyan; H, white; N, blue; O, red. Cα carbons are represented by blue spheres.

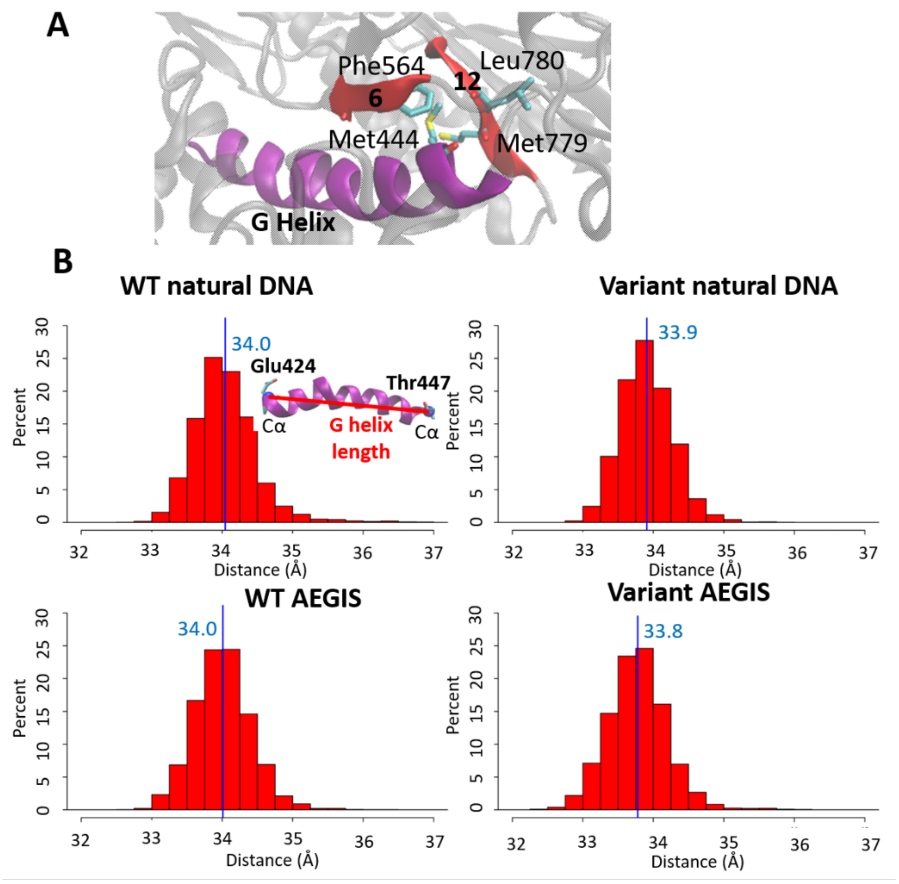

Figure S10: Hydrogen bond interactions involving the substituted residues (red) (A) M444V and (B) D551E. The protein structure is in cartoon coloured in grey and the residues in liquorice and the atoms in named residues are colored according to the scheme: C, cyan; H, white; N, blue; O, red; S, yellow. The hydrogen bonds (≥ 10% occurrence) are represented by black dashed lines and their percentage of occurrence is shown in black.

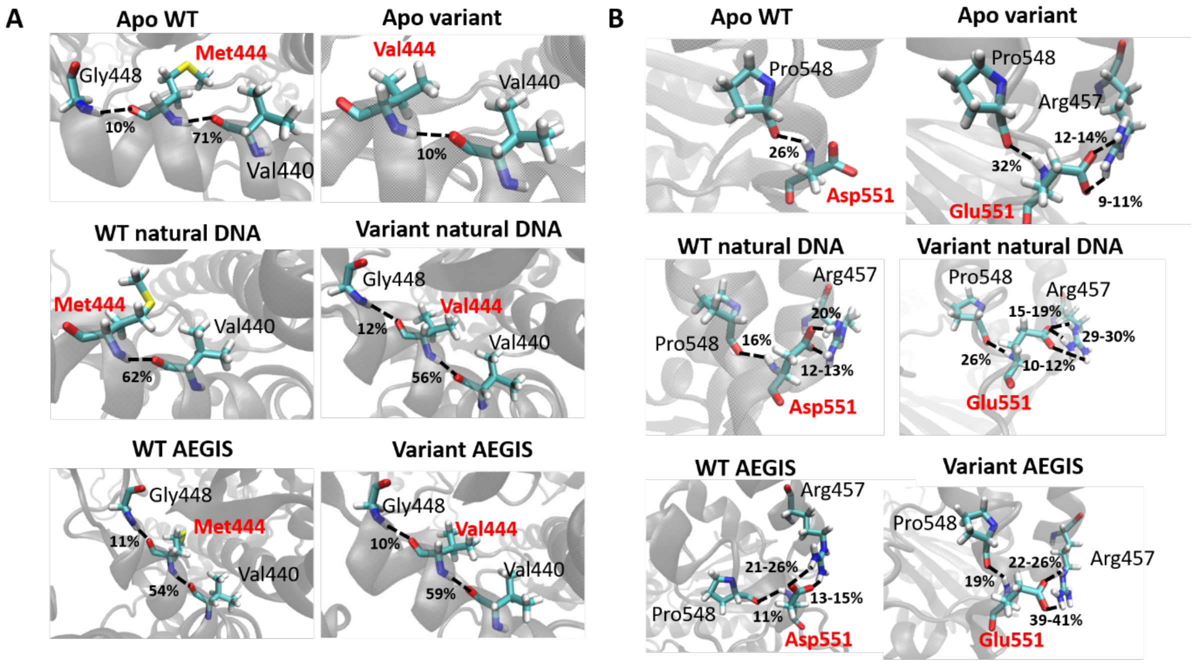

Figure S11: Root mean square deviation (RMSD) of the Cα positions for each simulated system relative to the equilibrated structure of the WT polymerase/Watson-Crick DNA binary complex, as calculated using CPPTRAJ in the AMBER 2016 software suite. An alignment of the reference structure (red) and the last frame of the MD simulation (blue) illustrate the stability of the system in the MD simulation.

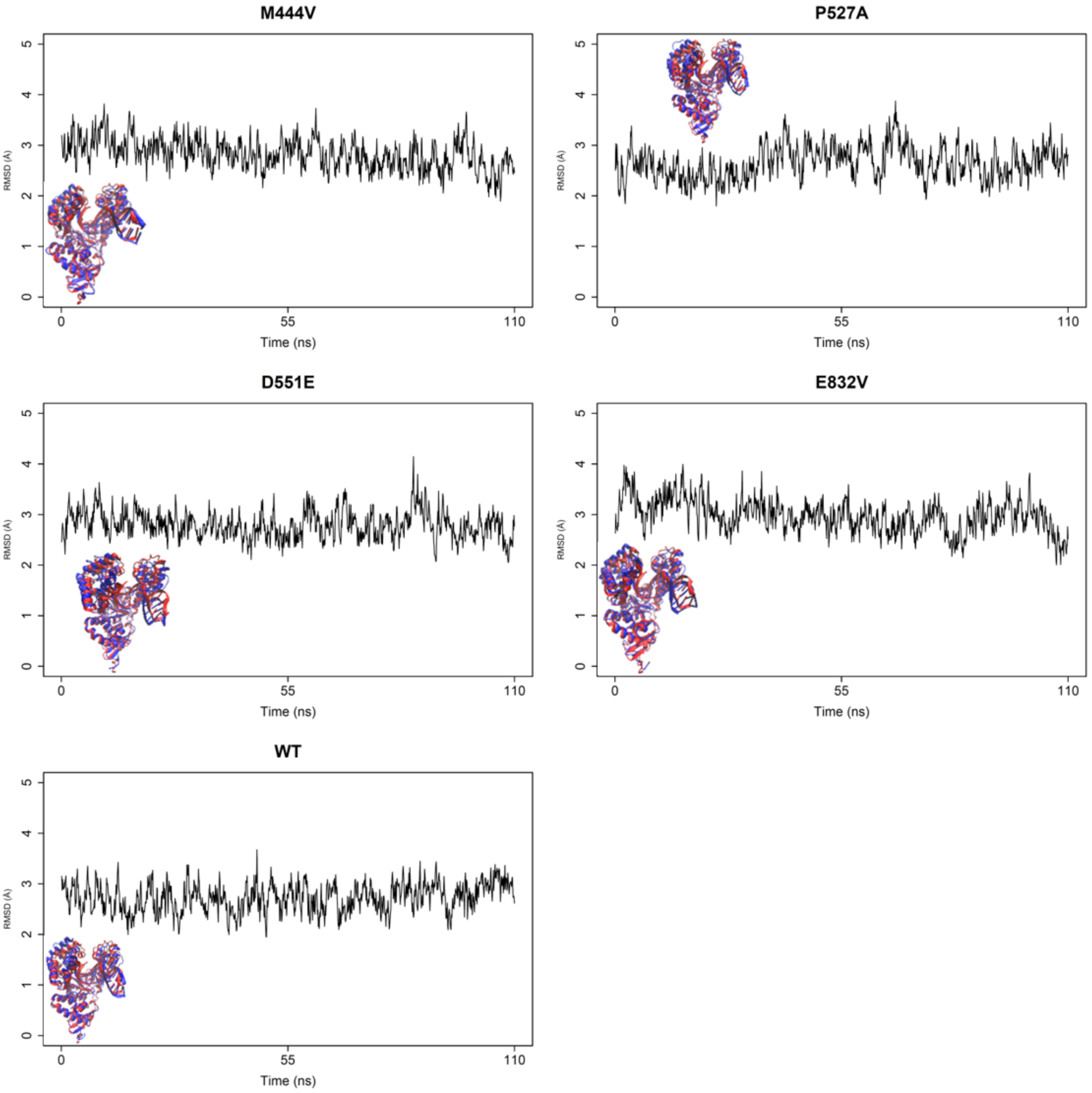

Figure S12: Hydrogen bonding interactions involving protein residues and the terminal nucleobase pair of the primer/template in the (top) WT polymerase/AEGIS DNA, (middle) P527A KlenTaq variant/AEGIS DNA, and (bottom) E832V KlenTaq variant/AEGIS DNA binary complexes. Atoms in the DNA and protein residues are colored according to the scheme: C, cyan; H, white; N, blue; O, red. Hydrogen bonds (≥ 30% occurrence during the sample MD trajectory) are represented by dashed lines and their percentage of occurrence is noted in black.

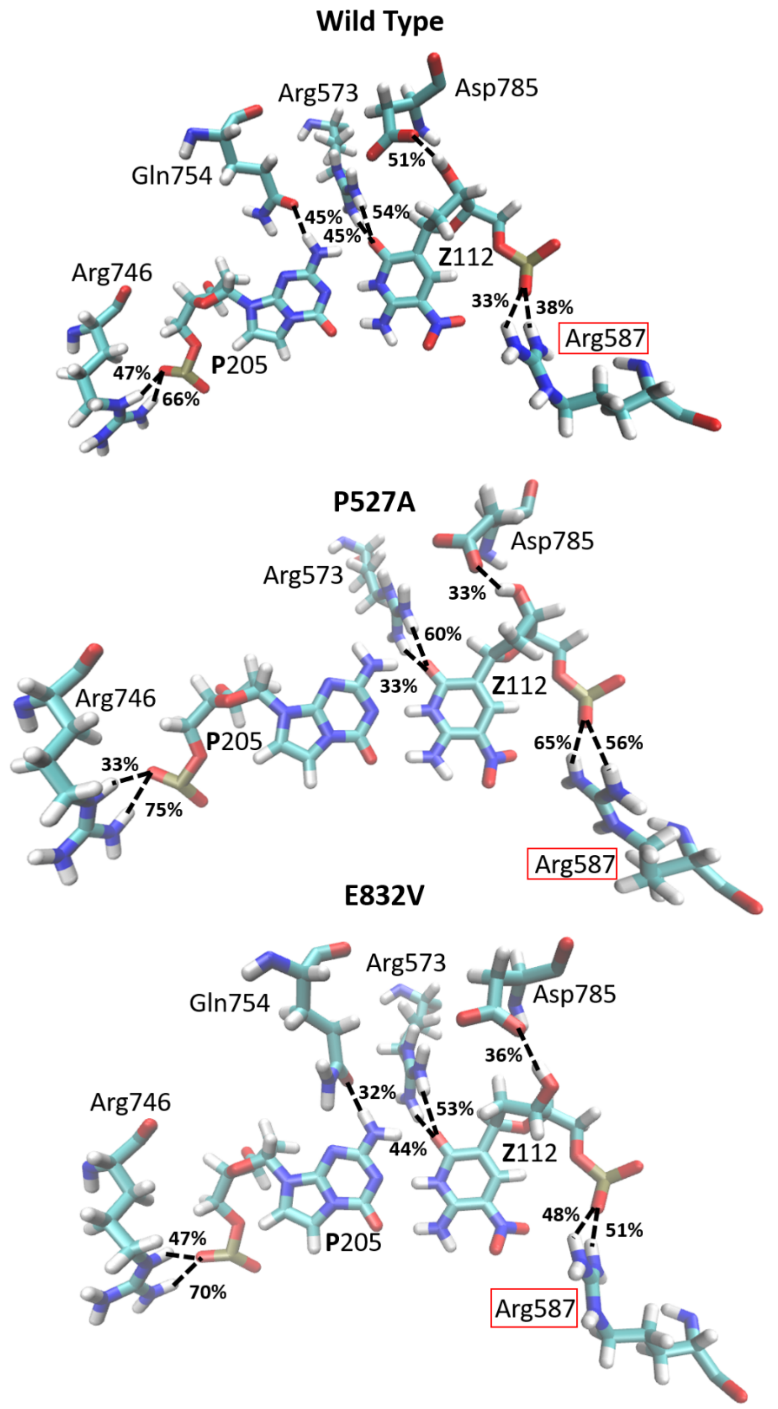

Figure S13: Dynamical cross correlation maps (DCCM) of the single variants P527A and E832V; (left/top triangle) P527A/AEGIS DNA binary complex, (left/bottom triangle) WT/AEGIS DNA binary complex, (right/top triangle) E832V/AEGIS DNA binary complex, (right/bottom triangle) WT/AEGIS DNA binary complex. Correlated (range: 0.25 to 1) and anti-correlated (range: -0.25 to -1) motions are colored from light to dark blue and pink, respectively. Areas rendered in white correspond to non-correlated motions (range: -0.25 to 0.25. Secondary structural elements are also included on the maps.

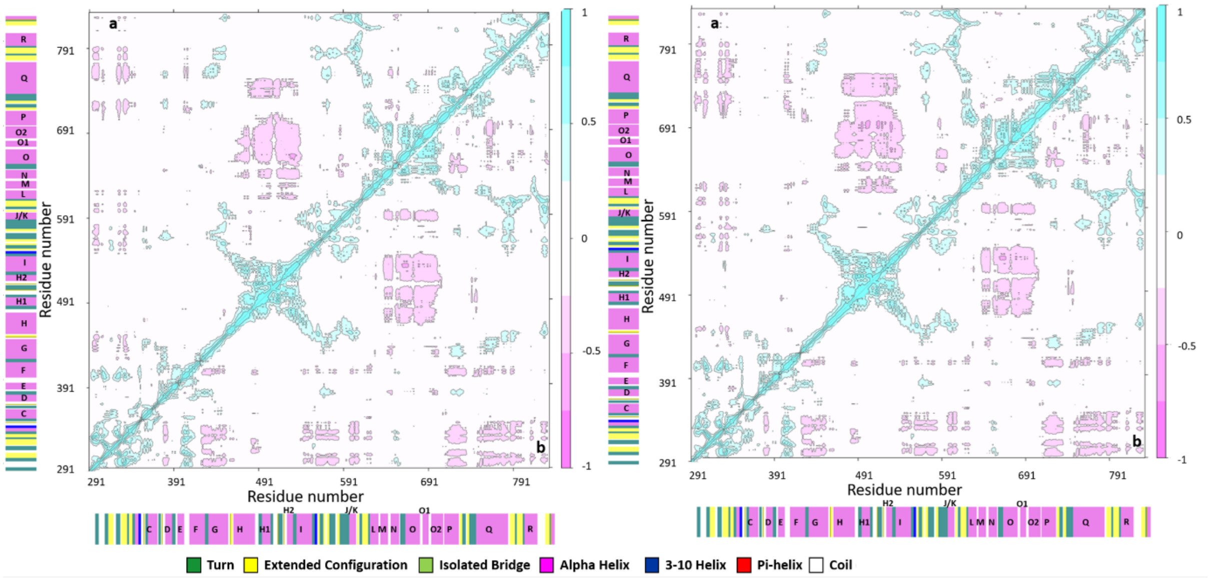

Supplementary Data S1: FRCMOD file entries for the **Z** and **P** AEGIS nucleobases.

Z, 6-amino-3-(2′-deoxyribofuranosyl)-5-nitro-1H-pyridin-2-one

```
remark goes here
MASS
O2 16.000        0.434              same as o
P  30.970        1.538              same as p4
OS 16.000        0.465              same as os
CI 12.010        0.000              ZO taken from parmbsc0
H1 1.008         0.135              same as hc
CT 12.010        0.878              same as c3
CE 12.001        0.000              ZO taken from parmbsc1
OH 16.000        0.465              same as oh
HO 1.008         0.135              same as ho
HC 1.008         0.135              same as hc
H2 1.008         0.135              same as hc
CM 12.010        0.360              same as c2
H4 1.008         0.135              same as ha
C  12.010        0.616              same as c
O  16.000        0.434              same as o
N* 14.010        0.530              same as na
H  1.008         0.161              same as hn
CA 12.010        0.360              same as c2
N2 14.010        0.530              same as n3

BOND
O2-P   525.00   1.480       ZO taken from parm10
P -OS  230.00   1.610       ZO taken from parm10
OS-CI  320.00   1.410       parmbsc0
CI-H1  340.00   1.090       parmbsc0
CI-CT  310.00   1.526       parmbsc0 same as CI-CT
CT-OS  320.00   1.410       ZO taken from parm10
CT-H1  340.00   1.090       ZO taken from parm10
CT-CE  310.00   1.526       parmbsc1 same as CE-CT
CE-OH  320.0    1.410       parmbsc1
CE-H1  340.00   1.090       parmbsc1
OH-HO  553.0    0.960       ZO taken from parm10 same as HO-OH
CT-HC  340.00   1.090       ZO taken from parm10
CT-CT  310.00   1.526       ZO taken from parm10
CT-H2  340.00   1.090       ZO taken from parm10
CT-CM  317.0    1.510       ZO taken from parm10 CM-CT
CM-CM  549.0    1.350       ZO taken from parm10 CM-CT
CM-C   410.0    1.444       ZO taken from parm10 C -CM
CM-H4  367.0    1.080       ZO taken from parm10
C -O   570.00   1.229       ZO same as C-O in parm10
C -N*  424.00   1.383       ZO taken from parm10
N*-H   434.0    1.010       ZO same as H -N* in parm10
N*-CA  397.40   1.353       ZO lenght taken from NWchem
CA-N2  481.00   1.340       ZO same as CA-N2 in parm10
CA-CM  427.0    1.433       ZO taken from parm10
N2-H   434.00   1.010       ZO same as N2-H in parm10
CM-no  364.20   1.423       ZO lenght taken from NWchem
no-o   550.00   1.214       ZO force constant taken between C-O and P-O2 and lenght taken from
NWChem

ANGLE
O2-P -O2  140.000     119.900   ZO taken from parm10
O2-P -OS  100.000     108.230   ZO taken from parm10
P -OS-CI  100.000     120.500   parmbsc0 P -OS-CI
OS-CI-H1   50.000     109.500   parmbsc0 OS-CI-H1
OS-CI-CT   50.000     109.500   parmbsc0 OS-CI-CT
CI-CT-OS   50.000     109.500   parmbsc0 CI-CT-OS
CI-CT-H1   50.0       109.50    parmbsc0 CI-CT-H1
CI-CT-CE   40.0       109.50    parmbsc1 CI-CT-CE
H1-CI-H1   35.000     109.500   parmbsc0
H1-CI-CT   50.000     109.500   parmbsc0 H1-CI-CT
CT-OS-CT   60.000     109.500   ZO taken from parm10
CT-CE-OH   50.0       109.50    parmbsc1
CT-CE-H1   50.0       109.50    parmbsc1
```

```
CT-CE-CT   40.000     109.500   parmbsc1
OS-CT-H1   50.000     109.500   ZO taken from parm10 H1-CT-OS
OS-CT-CE   50.000     109.500   parmbsc1 CE-CT-OS
OS-CT-CT   50.000     109.500   ZO taken from parm10 CT-CT-OS
OS-CT-H2   50.000     109.500   ZO taken from parm10 H2-CT-OS
OS-CT-CM   50.0       109.50    ZO taken from parm10 CM-CT-OS
H1-CT-CE   50.000     109.500   parmbsc1
CE-OH-HO   55.0       108.50    parmbsc1
CE-CT-HC   50.0       109.50    parmbsc1
CE-CT-CT   40.0       109.50    parmbsc1
OH-CE-H1   50.0       109.50    parmbsc1
CT-CT-H2   50.000     109.500   ZO taken from parm10 CT-CT-H2
CT-CT-CM   63.0       111.00    ZO taken from parm10 CM-CT-CT
HC-CT-HC   35.000     109.500   ZO taken from parm10
HC-CT-CT   50.000     109.500   ZO taken from parm10
CT-CM-CM   64.060     123.625   ZO angle taken from NWchem
CT-CM-C    70.0       119.70    ZO taken from parm10 C -CM-CT
H2-CT-CM   46.990     108.837   ZO angle taken from NWchem
CM-CM-H4   50.0       119.70    ZO taken from parm10
CM-CM-CM   67.940     122.666   ZO angle taken from NWchem
CM-C -O    80.0       125.30    ZO taken from parm10
CM-C -N*   70.0       114.834   ZO parm10 CM-N*-CT correct one not found and angle taken from
NWchem
CM-CM-C    63.0       120.70    ZO taken from parm10 C -CM-CM
CM-CM-CA   63.0       117.00    ZO taken from parm10 CA-CM-CM
CM-CM-no   70.070     118.913   ZO angle taken from NWchem
C -N*-H    50.0       119.20    ZO taken from parm10
C -N*-CA   70.0       121.60    ZO parm10 C -N*-CM correct one not found
O -C -N*   80.0       120.90    ZO taken from parm10 N*-C -O
N*-CA-N2   73.151     115.438   ZO angle taken from NWchem
N*-CA-CM   69.140     116.557   ZO angle taken from NWchem
H -N*-CA   47.300     119.057   ZO angle taken from NWchem
CA-N2-H    50.0       120.00    ZO taken from parm10
CA-CM-no   70.070     121.596   ZO angle taken from NWchem
N2-CA-CM   70.0       120.10    ZO taken from parm10
H -N2-H    35.000     120.000   ZO taken from parm10
CM-no-o    70.130     118.698   ZO angle taken from NWchem
o -no-o   116.649     125.080   ZO taken from gaff angle from NWchem

DIHE
O2-P -OS-CI   3    0.750         0.000          3.000      ZO taken from parm10 X -OS-P -X
P -OS-CI-H1   3    1.150         0.000          3.000      parmbsc0 X -CI-OS-X
P -OS-CI-CT   3    1.150         0.000          3.000      parmbsc0 X -CI-OS-X
OS-CI-CT-OS   9    1.400         0.000          3.000      parmbsc0 X -CI-CT-X
OS-CI-CT-H1   1    0.250         0.000          1.000      parmbsc0 H1-CT-CI-OS
OS-CI-CT-CE   1    1.178       190.976         -1.000      parmbsc1 CE-CT-CI-OS
OS-CI-CT-CE   1    0.092       295.632         -2.000      parmbsc1 CE-CT-CI-OS
OS-CI-CT-CE   1    0.962       348.095          3.000      parmbsc1 CE-CT-CI-OS
CI-CT-OS-CT   1    0.383         0.000         -3.000      parmbsc0 CT-OS-CT-CI
CI-CT-CE-OH   9    1.400         0.0            3.0        parmbsc1 X -CE-CT-X
CI-CT-CE-H1   9    1.400         0.000          3.000      parmbsc1 X -CE-CT-X
CI-CT-CE-CT   9    1.400         0.000          3.000      parmbsc1 X -CE-CT-X
H1-CI-CT-OS   1    0.250         0.000          1.000      parmbsc0 H1-CI-CT-OS
H1-CI-CT-H1   9    1.400         0.000          3.000      parmbsc0 X -CI-CT-X
H1-CI-CT-CE   9    1.400         0.000          3.000      parmbsc0 X -CI-CT-X
CT-OS-CT-CT   1    0.383         0.000         -3.000      ZO taken from parm10 CT-CT-OS-CT
CT-OS-CT-CT   1    0.100       180.000          2.000      ZO taken from parm10 CT-CT-OS-CT
CT-OS-CT-H2   3    1.150         0.000          3.000      ZO taken from parm10 X -CT-OS-X
CT-OS-CT-CM   3    1.150         0.000          3.000      ZO taken from parm10 X -CT-OS-X
CT-CE-OH-HO   1    1.079933    297.76503       -1.0        parmbsc1 HO-OH-CE-CT epsilon,
3'term
CT-CE-OH-HO   1    0.319017    200.61045       -2.0        parmbsc1 HO-OH-CE-CT epsilon,
3'term
CT-CE-OH-HO   1    0.054836    151.62658        3.0        parmbsc1 HO-OH-CE-CT epsilon,
3'term
CT-CE-CT-HC   9    1.400         0.0            3.0        parmbsc1 X -CE-CT-X
CT-CE-CT-CT   1    1.2          38.45          -1.0        parmbsc1 CT-CT-CE-CT
CT-CE-CT-CT   1    0.9          -2.23           3.0        parmbsc1 CT-CT-CE-CT
OS-CT-CE-OH   1    0.144         0.0           -3.0        parmbsc1 OH-CE-CT-OS
OS-CT-CE-OH   1    1.175         0.0            2.0        parmbsc1 OH-CE-CT-OS
OS-CT-CE-H1   9    1.400         0.0            3.0        parmbsc1 X -CE-CT-X
OS-CT-CE-CT   9    1.400         0.0            3.0        parmbsc1 X -CE-CT-X
OS-CT-CT-CE   9    1.40          0.0            3.         ZO parm10 X -CT-CT-X
OS-CT-CT-HC   1    0.000         0.000         -3.000      ZO taken from parm10 HC-CT-CT-OS
OS-CT-CT-HC   1    0.250         0.000          1.000      ZO taken from parm10 HC-CT-CT-OS
OS-CT-CM-CM   6    0.00          0.0            3.         ZO taken from parm10 X -CM-CT-X
OS-CT-CM-C    6    0.00          0.0            3.         ZO taken from parm10 X -CM-CT-X
```

```
H1-CT-OS-CT   3    1.150         0.000          3.000      ZO taken from parm10 X -CT-OS-X
H1-CT-CE-OH   9    1.400         0.0            3.0        parmbsc1 X -CE-CT-X
H1-CT-CE-H1   9    1.400         0.0            3.0        parmbsc1 X -CE-CT-X
H1-CT-CE-CT   9    1.400         0.0            3.0        parmbsc1 X -CE-CT-X
CE-CT-OS-CT   1    0.3833        0.0           -3.0        parmbsc1
CE-CT-OS-CT   1    0.100       180.0            2.0        parmbsc1
CE-CT-CT-H2   9    1.40          0.0            3.         ZO parm10 X -CT-CT-X
CE-CT-CT-CM   9    1.40          0.0            3.         ZO parm10 X -CT-CT-X
OH-CE-CT-HC   9    1.400         0.0            3.0        parmbsc1 X -CE-CT-X
OH-CE-CT-CT   9    1.400         0.0            3.0        parmbsc1 X -CE-CT-X
HO-OH-CE-H1   3    0.500         0.0            3.0        parmbsc1 X -CE-OH-X
H1-CE-CT-HC   9    1.400         0.0            3.0        parmbsc1 X -CE-CT-X
H1-CE-CT-CT   9    1.400         0.0            3.0        parmbsc1 X -CE-CT-X
CT-CT-CM-CM   6    0.00          0.0            3.         ZO parm10 X -CM-CT-X
CT-CT-CM-C    6    0.00          0.0            3.         ZO parm10 X -CM-CT-X
HC-CT-CT-H2   9    1.400         0.000          3.000      ZO taken from parm10 X -CT-CT-X
HC-CT-CT-CM   9    1.40          0.0            3.         ZO parm10 X -CT-CT-X
CT-CM-CM-H4   4   26.60        180.0            2.         ZO parm10 X -CM-CM-X
CT-CM-CM-CM   4   26.60        180.0            2.         ZO parm10 X -CM-CM-X
CT-CM-C -O    4    8.70        180.0            2.         ZO parm10 X -C -CM-X
CT-CM-C -N*   4    8.70        180.0            2.         ZO parm10 X -C -CM-X
H2-CT-CM-CM   6    0.00          0.0            3.         ZO parm10 X -CM-CT-X
H2-CT-CM-C    6    0.00          0.0            3.         ZO parm10 X -CM-CT-X
CM-CM-CM-CA   4   26.60        180.0            2.         ZO parm10 X -CM-CM-X
CM-CM-CM-no   4   26.60        180.0            2.         ZO parm10 X -CM-CM-X
CM-C -N*-H    4    5.80        180.0            2.         ZO parm10 X -C -N*-X
CM-C -N*-CA   4    5.80        180.0            2.         ZO parm10 X -C -N*-X
CM-CM-C -O    1    2.175       180.0           -2.         ZO parm10 CM-CM-C -O
CM-CM-C -O    1    0.30          0.0            3.         ZO parm10 CM-CM-C -O
CM-CM-C -N*   4    8.70        180.0            2.         ZO parm10 X -C -CM-X
CM-CM-CA-N*   4   10.20        180.0            2.         ZO parm10 X -CA-CM-X
CM-CM-CA-N2   4   10.20        180.0            2.         ZO parm10 X -CA-CM-X
CM-CM-no-o    1    0.750       180.000          2.000      same as X -c2-no-X
H4-CM-CM-C    4   26.60        180.0            2.         ZO parm10 X -CM-CM-X
H4-CM-CM-CA   4   26.60        180.0            2.         ZO parm10 X -CM-CM-X
H4-CM-CM-no   4   26.60        180.0            2.         ZO parm10 X -CM-CM-X
C -CM-CM-CM   4   26.60        180.0            2.         ZO parm10 X -CM-CM-X
C -N*-CA-N2   1    0.625       180.000          2.000      same as X -c2-na-X
C -N*-CA-CM   1    0.625       180.000          2.000      same as X -c2-na-X
O -C -N*-H    4    5.80        180.0            2.         ZO parm10 X -C -N*-X
O -C -N*-CA   4    5.80        180.0            2.         ZO parm10 X -C -N*-X
N*-CA-N2-H    4    9.60        180.0            2.         ZO parm10 X -CA-N2-X
N*-CA-CM-no   4   10.20        180.0            2.         ZO parm10 X -CA-CM-X
H -N*-CA-N2   1    0.625       180.000          2.000      same as X -c2-na-X
H -N*-CA-CM   1    0.625       180.000          2.000      same as X -c2-na-X
CA-CM-no-o    1    0.750       180.000          2.000      same as X -c2-no-X
N2-CA-CM-no   4   10.20        180.0            2.         ZO parm10 X -CA-CM-X
H -N2-CA-CM   4    9.60        180.0            2.         ZO parm10 X -CA-N2-X

IMPROPER
C -CM-CM-CT         5.6          180.         2.0          ZO parm10 C -CM-CM-CT
CM-CM-CM-H4         5.6          180.0        2.0          ZO 5.6
CM-N*-C -O          5.6          180.0        2.0          ZO 5.6
C -CA-N*-H          5.6          180.0        2.0          ZO 5.6
CM-N*-CA-N2         5.6          180.0        2.0          ZO 5.6
CA-H -N2-H          5.6          180.0        2.0          ZO 5.6
CA-CM-CM-no         5.6          180.0        2.0          ZO 5.6
CM-o -no-o          5.6          180.0        2.0          ZO 5.6

NONBON
  O2         1.6612  0.2100            same as o
  P          2.1000  0.2000            same as p4
  OS         1.6837  0.1700            same as os
  CI         1.9080  0.1094            ZO parmbsc0
  H1         1.3870  0.0157            ZO parm10 H1
  CT         1.9080  0.1094            same as c3
  CE         1.9080  0.1094            ZO parmbsc1
  OH         1.7210  0.2104            same as oh
  HO         0.0000  0.0000            same as ho
  HC         1.4870  0.0157            same as hc
  H2         1.2870  0.0157            ZO parm10 H2
  CM         1.9080  0.0860            ZO parm14 CM
  H4         1.4090  0.0150            ZO parm10 H4
  C          1.9080  0.0860            same as c
  O          1.6612  0.2100            same as o
  N*         1.8240  0.1700            ZO parm14 N*
  H          0.6000  0.0157            ZO parm10 H
```

```
  CA          1.9080  0.0860             ZO parm14 CA
  N2          1.8240  0.1700             ZO parm14 N2
```

P, 2-amino-8-(1-beta-D-2′-deoxyribofuranosyl)imidazo [1,2-a]-1,3,5-triazin-[8H]-4-one

```
remark goes here
MASS
O  16.000        0.434                same as o
C  12.010        0.616                same as c
NC 14.010        0.530                same as n2
CA 12.010        0.360                same as c2
N2 14.010        0.530                same as n3
H  1.008         0.161                same as hn
CB 12.010        0.360                same as c2
N* 14.010        0.530                same as na
CK 12.010        0.360                same as c2
H5 1.008         0.135                same as ha
CT 12.010        0.878                same as c3
OS 16.000        0.465                same as os
H2 1.008         0.135                same as hc
HC 1.008         0.135                same as hc
CE 12.001                             parmbsc1
H1 1.008         0.135                same as hc
CI 12.010                             parmbsc0
P  30.970        1.538                same as p4
O2 16.000        0.434                same as o

BOND
O -C   570.00   1.229       ZO same as C-O in parm10
C -NC  457.00   1.358       ZO same as C-NC in parm10
C -N*  424.00   1.383       ZO taken from parm10
NC-CA  483.00   1.339       ZO same as CA-NC in parm10
CA-N2  481.00   1.340       ZO same as CA-N2 in parm10
N2-H   434.00   1.010       ZO same as N2-H in parm10
CB-NC  461.00   1.354       ZO same as CB-NC in parm10
CB-N*  436.00   1.374       ZO same as CB-N* in parm10
N*-CK  440.00   1.371       ZO taken from parm10
CK-H5  367.00   1.080       ZO taken from parm10
CK-CK  419.80   1.428       same as cc-cc
N*-CT  337.00   1.475       ZO taken from parm10
CT-OS  320.00   1.410       ZO taken from parm10
CT-H2  340.00   1.090       ZO taken from parm10
CT-CT  310.00   1.526       ZO taken from parm10
CT-HC  340.00   1.090       ZO taken from parm10
CT-CE  310.00   1.526       parmbsc1 same as CE-CT
CE-OS  320.00   1.410       parmbsc1 same as OS-CE
CE-H1  340.00   1.090       parmbsc1
CT-H1  340.00   1.090       ZO taken from parm10
CT-CI  310.00   1.526       parmbsc0 same as CI-CT
CI-H1  340.00   1.090       parmbsc0
CI-OS  320.00   1.410       parmbsc0
OS-P   230.00   1.610       ZO taken from parm10
P -O2  525.00   1.480       ZO taken from parm10

ANGLE
O -C -NC   80.000     122.500   ZO taken from parm10 NC-C -O
O -C -N*   80.000     120.900   ZO taken from parm10 N*-C -O
C -NC-CA   70.000     120.500   ZO taken from parm10
C -N*-CB   70.000     121.600   ZO taken from parm10 C -N*-CM
C -N*-CK   70.000     121.600   ZO taken from parm10 C -N*-CM
NC-C -N*   70.000     118.600   ZO taken from parm10 N*-C -NC
NC-CA-N2   70.000     119.300   ZO taken from parm10 N2-CA-NC
NC-CA-NC   69.850     128.740   same as nc-ca-nc
CA-N2-H    50.000     120.000   ZO taken from parm10
CA-NC-CB   70.000     112.200   ZO taken from parm10
H -N2-H    35.000     120.000   ZO taken from parm10
NC-CB-N*   70.000     126.200   ZO taken from parm10 N*-CB-NC
CB-N*-CK   70.000     105.400   ZO taken from parm10
CB-N*-CT   70.000     125.800   ZO taken from parm10
N*-CB-N*   70.000     113.900   ZO taken from parm10 N*-C5-NB
N*-CK-H5   50.000     123.050   ZO taken from parm10 H5-CK-N*
N*-CK-CK   86.536     117.770   ZO taken from gaff2 cc-cc-na
CK-CK-H5   47.634     121.070   ZO taken from gaff2 cc-cc-ha
CK-N*-CT   70.000     128.800   ZO taken from parm10
N*-CT-OS   50.000     109.500   ZO taken from parm10 OS-CT-N*
```

```
N*-CT-H2   50.000     109.500   ZO taken from parm10 H2-CT-N*
N*-CT-CT   50.000     109.500   ZO taken from parm10 CT-CT-N*
CT-OS-CT   60.000     109.500   ZO taken from parm10
CT-CT-HC   50.000     109.500   ZO taken from parm10
CT-CT-CE   40.000     109.500   parmbsc1 CE-CT-CT
OS-CT-H2   50.000     109.500   ZO taken from parm10 H2-CT-OS
OS-CT-CT   50.000     109.500   ZO taken from parm10 CT-CT-OS
OS-CT-CE   50.000     109.500   parmbsc1 CE-CT-OS
OS-CT-H1   50.000     109.500   ZO taken from parm10 H1-CT-OS
OS-CT-CI   50.000     109.500   parmbsc0 CI-CT-OS
H2-CT-CT   50.000     109.500   ZO taken from parm10 CT-CT-H2
CT-CE-OS   50.000     109.500   parmbsc1 OS-CE-CT
CT-CE-H1   50.000     109.500   parmbsc1 H1-CE-CT
CT-CE-CT   40.000     109.500   parmbsc1
HC-CT-HC   35.000     109.500   ZO taken from parm10
HC-CT-CE   50.000     109.500   parmbsc1 CE-CT-HC
CE-CT-H1   50.000     109.500   parmbsc1
CE-CT-CI   40.000     109.500   parmbsc1 CI-CT-CE
OS-CE-H1   50.000     109.500   parmbsc1
CT-CI-H1   50.000     109.500   parmbsc0 H1-CI-CT
CT-CI-OS   50.000     109.500   parmbsc0 OS-CI-CT
H1-CT-CI   50.000     109.500   parmbsc0 CI-CT-H1
CI-OS-P   100.000     120.500   parmbsc0 P -OS-CI
H1-CI-H1   35.000     109.500   parmbsc0
H1-CI-OS   50.000     109.500   parmbsc0 OS-CI-H1
OS-P -O2  100.000     108.230   ZO taken from parm10 O2-P -OS
O2-P -O2  140.000     119.900   ZO taken from parm10

DIHE
O -C -NC-CA   2    8.000      180.000          2.000     ZO taken from parm10 X -C -NC-X
O -C -N*-CB   4    5.800      180.000          2.000     ZO taken from parm10 X -C -N*-X
O -C -N*-CB   4    5.800      180.000          2.000     ZO taken from parm10 X -C -N*-X
O -C -N*-CK   4    5.800      180.000          2.000     ZO taken from parm10 X -C -N*-X
O -C -N*-CK   4    5.800      180.000          2.000     ZO taken from parm10 X -C -N*-X
C -NC-CA-N2   4   14.500      180.000          2.000     ZO taken from parm10 X -CA-CN-X
C -NC-CA-NC   4   14.500      180.000          2.000     ZO taken from parm10 X -CA-CN-X
C -N*-CB-NC   4    6.600      180.000          2.000     ZO taken from parm10 X -CB-N*-X
C -N*-CB-N*   4    6.600      180.000          2.000     ZO taken from parm10 X -CB-N*-X
C -N*-CK-H5   4    6.800      180.000          2.000     ZO taken from parm10 X -CK-N*-X
C -N*-CK-CK   4    6.800      180.000          2.000     ZO taken from parm10 X -CK-N*-X
NC-C -N*-CB   4    5.800      180.000          2.000     ZO taken from parm10 X -C -N*-X
NC-C -N*-CB   4    5.800      180.000          2.000     ZO taken from parm10 X -C -N*-X
NC-C -N*-CK   4    5.800      180.000          2.000     ZO taken from parm10 X -C -N*-X
NC-C -N*-CK   4    5.800      180.000          2.000     ZO taken from parm10 X -C -N*-X
NC-CA-N2-H    4    9.600      180.000          2.000     ZO taken from parm10 X -CA-N2-X
NC-CA-NC-CB   2    9.600      180.000          2.000     ZO taken from parm10 X -CA-NC-X
CA-NC-C -N*   2    8.000      180.000          2.000     ZO taken from parm10 X -C -NC-X
CA-NC-CB-N*   2    8.300      180.000          2.000     ZO taken from parm10 X -CB-NC-X
N2-CA-NC-CB   4   14.500      180.000          2.000     ZO taken from parm10 X -CA-CN-X
NC-CB-N*-CK   4    6.600      180.000          2.000     ZO taken from parm10 X -CB-N*-X
NC-CB-N*-CT   4    6.600      180.000          2.000     ZO taken from parm10 X -CB-N*-X
CB-N*-CK-H5   4    6.800      180.000          2.000     ZO taken from parm10 X -CK-N*-X
CB-N*-CK-CK   4    6.800      180.000          2.000     ZO taken from parm10 X -CK-N*-X
CB-N*-CT-OS   1    0.000      000.000         -2.000     ZO taken from parm10 OS-CT-N*-CK
CB-N*-CT-OS   1    2.500        0.000          1.000     ZO taken from parm10 OS-CT-N*-CK
CB-N*-CT-H2   6    0.000        0.000          2.000     ZO taken from parm10 X -CT-N*-X
CB-N*-CT-CT   6    0.000        0.000          2.000     ZO taken from parm10 X -CT-N*-X
N*-CB-N*-CK   4    6.600      180.000          2.000     ZO taken from parm10 X -CB-N*-X
N*-CB-N*-CT   4    6.600      180.000          2.000     ZO taken from parm10 X -CB-N*-X
N*-CK-CK-H5   4   16.000      180.000          2.000     ZO taken from gaff2 X -cc-cc-X
N*-CK-CK-N*   4   16.000      180.000          2.000     ZO taken from gaff2 X -cc-cc-X
CK-CK-N*-CT   4    6.800      180.000          2.000     ZO taken from parm10 X -CK-N*-X
H5-CK-CK-H5   4   16.000      180.000          2.000     ZO taken from gaff2 X -cc-cc-X
CK-N*-CT-OS   1    0.000      000.000         -2.000     ZO taken from gaff2 OS-CT-N*-CK
CK-N*-CT-OS   1    2.500        0.000          1.000     ZO taken from gaff2 OS-CT-N*-CK
CK-N*-CT-H2   6    0.000        0.000          2.000     ZO taken from parm10 X -CT-N*-X
CK-N*-CT-CT   6    0.000        0.000          2.000     ZO taken from parm10 X -CT-N*-X
H5-CK-N*-CT   4    6.800      180.000          2.000     ZO taken from parm10 X -CK-N*-X
N*-CT-OS-CT   1    0.383        0.000         -3.000     ZO taken from parm10 CT-OS-CT-N*
N*-CT-OS-CT   1    0.650        0.000          2.000     ZO taken from parm10 CT-OS-CT-N*
N*-CT-CT-HC   9    1.400        0.000          3.000     ZO taken from parm10 X -CT-CT-X
N*-CT-CT-CE   9    1.400        0.000          3.000     ZO taken from parm10 X -CT-CT-X
CT-OS-CT-CE   1    0.383        0.000         -3.000     parmbsc1 CE-CT-OS-CT
CT-OS-CT-CE   1    0.100      180.000          2.000     parmbsc1 CE-CT-OS-CT
CT-OS-CT-H1   3    1.150        0.000          3.000     ZO taken from parm10 X -CT-OS-X
CT-OS-CT-CI   1    0.383        0.000         -3.000     parmbsc0 CT-OS-CT-CI
CT-CT-CE-OS   9    1.400        0.000          3.000     parmbsc1 X -CE-CT-X
```

```
CT-CT-CE-H1   9    1.400         0.000          3.000      parmbsc1 X -CE-CT-X
CT-CT-CE-CT   1    1.200        38.450         -1.000      parmbsc1 CT-CT-CE-CT
CT-CT-CE-CT   1    0.900        -2.230          3.000      parmbsc1 CT-CT-CE-CT
OS-CT-CT-HC   1    0.000         0.000         -3.000      ZO taken from parm10 HC-CT-CT-OS
OS-CT-CT-HC   1    0.250         0.000          1.000      ZO taken from parm10 HC-CT-CT-OS
OS-CT-CT-CE   9    1.400         0.000          3.000      ZO taken from parm10 X -CT-CT-X
OS-CT-CE-CT   1    1.200       322.410          1.000      parmbsc1 CT-CE-CT-OS
OS-CT-CE-OS   1    0.144         0.000         -3.000      parmbsc1 OS-CE-CT-OS
OS-CT-CE-OS   1    1.175         0.000          2.000      parmbsc1 OS-CE-CT-OS
OS-CT-CE-H1   9    1.400         0.000          3.000      parmbsc1 X -CE-CT-X
OS-CT-CI-H1   1    0.250         0.000          1.000      parmbsc0 H1-CI-CT-OS
OS-CT-CI-OS   9    1.400         0.000          3.000      parmbsc0 X -CI-CT-X
H2-CT-OS-CT   3    1.150         0.000          3.000      ZO taken from parm10 X -CT-OS-X
H2-CT-CT-HC   9    1.400         0.000          3.000      ZO taken from parm10 X -CT-CT-X
H2-CT-CT-CE   9    1.400         0.000          3.000      ZO taken from parm10 X -CT-CT-X
CT-CT-OS-CT   1    0.383         0.000         -3.000      ZO taken from parm10 CT-CT-OS-CT
CT-CT-OS-CT   1    0.100       180.000          2.000      ZO taken from parm10 CT-CT-OS-CT
CT-CE-CT-H1   9    1.400         0.000          3.000      parmbsc1 X -CE-CT-X
CT-CE-CT-CI   9    1.400         0.000          3.000      parmbsc1 X -CE-CT-X
HC-CT-CE-OS   9    1.400         0.000          3.000      parmbsc1 X -CE-CT-X
HC-CT-CE-H1   9    1.400         0.000          3.000      parmbsc1 X -CE-CT-X
HC-CT-CE-CT   9    1.400         0.000          3.000      parmbsc1 X -CE-CT-X
CE-CT-CI-H1   9    1.400         0.000          3.000      parmbsc0 X -CI-CT-X
CE-CT-CI-OS   1    1.178       190.976         -1.000      parmbsc1 CE-CT-CI-OS
CE-CT-CI-OS   1    0.092       295.632         -2.000      parmbsc1 CE-CT-CI-OS
CE-CT-CI-OS   1    0.962       348.095          3.000      parmbsc1 CE-CT-CI-OS
OS-CE-CT-H1   9    1.400         0.000          3.000      parmbsc1 X -CE-CT-X
OS-CE-CT-CI   9    1.400         0.000          3.000      parmbsc1 X -CE-CT-X
H1-CE-CT-H1   9    1.400         0.000          3.000      parmbsc1 X -CE-CT-X
H1-CE-CT-CI   9    1.400         0.000          3.000      parmbsc1 X -CE-CT-X
CT-CI-OS-P    3    1.150         0.000          3.000      parmbsc0 X -CI-OS-X
H1-CT-CI-H1   9    1.400         0.000          3.000      parmbsc0 X -CI-CT-X
H1-CT-CI-OS   1    0.250         0.000          1.000      parmbsc0 H1-CT-CI-OS
CI-OS-P -O2   3    0.750         0.000          3.000      ZO taken from parm10 X -OS-P -X
H1-CI-OS-P    3    1.150         0.000          3.000      parmbsc0 X -CI-OS-X

IMPROPER
N*-NC-C -O         10.5          180.0        2.0          ZO taken from parm10 X -X -C -O
N2-NC-CA-NC         5.6          180.0        2.0          Using default value
CA-H -N2-H          5.6          180.0        2.0          Using default value
N*-N*-CB-NC         5.6          180.0        2.0          Using default value
C -CB-N*-CK         5.6          180.0        2.0          Using default value
CK-H5-CK-N*         5.6          180.0        2.0          Using default value
CB-CK-N*-CT         5.6          180.0        2.0          ZO taken from parm10 CB-CK-N*-CT
N*-CB-CB-NC         5.6          180.0        2.0          added after tleap complain
NB-C-CB-CB          5.6          180.0        2.0          added after tleap complain
N*-CB-CB-NC         5.6          180.0        2.0          added after tleap complain
NB-C-CB-CB          5.6          180.0        2.0          added after tleap complain
NB-CA-CB-CB         5.6          180.0        2.0          added after tleap complain

NONBON
  O          1.6612  0.2100            same as o in parm10
  C          1.9080  0.0860            same as c in parm10
  NC         1.8240  0.1700            same as nc in parm14
  CA         1.9080  0.0860            same as ca in parm14
  N2         1.8240  0.1700            same as nh in parm14
  H          0.6000  0.0157            same as hn in parm14
  CB         1.9080  0.0860            same as cc in parm14
  N*         1.8240  0.1700            same as N* in parm14
  CK         1.9080  0.0860            same as CK in parm14
  H5         1.3590  0.0150            ZO taken from parm10 H5
  CT         1.9080  0.1094            same as c3 in parm10
  OS         1.6837  0.1700            same as os in parm10
  H2         1.2870  0.0157            ZO taken from parm10 H2
  HC         1.4870  0.0157            same as hc in parm10
  CE         1.9080  0.1094            parmbsc1
  H1         1.3870  0.0157            ZO taken from parm10 H1
  CI         1.9080  0.1094            parmbsc0
  P          2.1000  0.2000            same as P in parm10
  O2         1.6612  0.2100            same as o  in parm10
```
